# Efficacy and safety of novel antidiabetic drugs in patients with type 2 diabetes and chronic kidney disease: a network meta-analysis

**DOI:** 10.3389/fendo.2026.1750615

**Published:** 2026-03-31

**Authors:** Xiaojian Zhu, Xingjia Wang, Peiru Zhang, Zhuoshi Yang, Na Zhao, Jiameng Li, Yunze Shi, Yichen Zhao, Jian Ma

**Affiliations:** 1 Graduate School, Heilongjiang University of Chinese Medicine, Harbin, Heilongjiang, China; 2 Department of Endocrinology, The First Affiliated Hospital of Heilongjiang University of Chinese Medicine, Harbin, Heilongjiang, China; 3 The First Clinical Medical College, Heilongjiang University of Chinese Medicine, Harbin, Heilongjiang, China

**Keywords:** antidiabetic drugs, cardiovascular outcomes, chronic kidney disease, meta-analysis, renal outcomes, type 2 diabetes

## Abstract

**Background:**

A number of novel antidiabetic drugs have been developed. These drugs include sodium-glucose cotransporter 2 inhibitors (SGLT-2is), glucagon-like peptide-1 receptor agonists (GLP-1RAs), and dipeptidyl peptidase-4 inhibitors (DPP-4is). However, the optimal medication for individuals with type 2 diabetes mellitus (T2DM) and comorbid chronic kidney disease (CKD) has not been established. To this end, this study was conducted to compare specific novel antidiabetic drugs regarding efficacy and safety.

**Methods:**

PubMed, Embase, Cochrane Library, and Web of Science databases were searched for publications dated as of July 9, 2025. Cochrane risk of bias tool version 2.0 (RoB 2.0) was applied to measure the quality of the publications, and R 4.2.2 and Stata 15.1 were used to execute a Bayesian network meta-analysis (NMA). Primary outcomes encompassed major adverse cardiovascular events (MACEs), composite renal outcomes, and all-cause mortality (ACM). Secondary outcomes comprised adverse events (AEs), hypoglycemia, and cardiovascular death.

**Results:**

This NMA incorporated 30 studies, involving 39,844 participants with T2DM and comorbid CKD. The interventions were ranked by performance in various outcomes using the surface under the cumulative ranking curve (SUCRA) values. Sotagliflozin ranked first in reducing MACEs (SUCRA: 90.57%). Empagliflozin ranked first in improving composite renal outcomes (SUCRA: 89.76%) and reducing ACM (SUCRA: 72.38%). Canagliflozin ranked first in reducing AEs (SUCRA: 83.37%). Dapagliflozin + exenatide ranked first in reducing hypoglycemic events (SUCRA: 77.74%). Semaglutide ranked first in reducing cardiovascular mortality (SUCRA: 89.46%).

**Conclusion:**

Novel antidiabetic drugs offer benefits for patients with T2DM and comorbid CKD. However, the optimal intervention varies for different outcomes. Further clinical studies are anticipated to validate these findings.

**Systematic Review Registration:**

https://www.crd.york.ac.uk/prospero/, identifier CRD420251146144.

## 1 Introduction

Type 2 diabetes mellitus (T2DM) complicated by chronic kidney disease (CKD) (T2DM-CKD) represents a crucial global health issue. With a constantly rising prevalence, it has imposed a huge burden on healthcare systems (1). Globally, the prevalence of CKD among T2DM patients is high (approximately 31.4%) (2). T2DM is the foremost contributor to end-stage renal disease (ESRD). The Global Burden of Disease Study 2021 revealed that 2.01 million people were newly diagnosed with T2DM-related CKD, showing a 150.92% increase since 1992. The age-standardized incidence was 15.09/100,000 in regions with low socioeconomic indexes and 23.07/100,000 in regions with high socioeconomic indexes, with the heaviest disease burden observed in Asia (1). CKD markedly elevates the risk of cardiovascular events through multiple interconnected pathways, including chronic inflammation, oxidative stress, endothelial dysfunction, and vascular calcification (3). Besides, it also leads to delayed treatment and increased healthcare costs due to inadequate monitoring (2). Therefore, early intervention and effective treatment are necessary.

Currently, the treatment of T2DM-CKD primarily involves comprehensive management strategies combining lifestyle intervention and drug therapy. International guidelines, such as the American Diabetes Association’s Standards of Care in Diabetes 2024, have recommended stratified management based on lifestyle modifications, combined with antidiabetic, antihypertensive, lipid-regulating, and renal protective medications (4). Some novel drugs have been proven to significantly reduce the risks of cardiovascular events and renal disease progression, particularly in individuals with concomitant atherosclerotic cardiovascular disease (ASCVD) or CKD (4). These drugs include sodium-glucose cotransporter 2 inhibitors (SGLT-2is), such as empagliflozin and dapagliflozin, and glucagon-like peptide-1 receptor agonists (GLP-1RAs), such as liraglutide and semaglutide. In addition, dipeptidyl peptidase-4 inhibitors (DPP-4is), such as sitagliptin and saxagliptin, improve glycemic control by prolonging the activation of endogenous GLP-1 and significantly reduce glycated hemoglobin (HbA1c) levels by approximately 0.4%-0.7% (5).

Previous systematic reviews and meta-analyses have primarily focused on comparisons among major classes of novel antidiabetic drugs (SGLT-2is, GLP-1RAs, and DPP-4is) (6, 7). However, comparisons between specific drugs are lacking. Consequently, this network meta-analysis (NMA) sought to synthesize as much information as possible from randomized controlled trials (RCTs) directly and indirectly. Various novel antidiabetic drugs were compared for safety and effectiveness in individuals with T2DM-CKD to offer additional clinical guidance.

## 2 Methods

This meta-analysis was conducted following the Preferred Reporting Items for Systematic Reviews and Meta-Analyses statement (8). The study protocol was registered in the International Prospective Register of Systematic Reviews (PROSPERO) (CRD420251146144).

### 2.1 Search strategy

PubMed, Embase, Cochrane Library, and Web of Science databases were searched for English publications dated from the establishment of each database to July 9, 2025. The following combinations of subject headings and free-text terms were used: diabetes mellitus type 2 and renal insufficiency, chronic and sodium-glucose transporter 2 inhibitors, dipeptidyl-peptidase 4 inhibitors, glucagon-like peptide 1, and randomized controlled trials. The search strategy employed is detailed in **Supplementary Table 1** of **Supplementary Material 1**. Additionally, the references of published systematic reviews were searched to ensure the most comprehensive coverage possible.

### 2.2 Inclusion and exclusion criteria

Articles meeting the following criteria were included in this study: (1) Study population: individuals with T2DM-CKD, covering stage 1 to stage 5 CKD (including patients with T2DM and ESRD receiving dialysis); (2) Interventions: novel antidiabetic drugs (SGLT-2is, DPP-4is, and GLP-1RAs); (3) Comparison: placebo or another drug distinct from the intervention; (4) Study design: RCT; (5) Outcomes: primary outcomes encompassed major adverse cardiovascular events (MACEs), composite renal outcomes, and all-cause mortality (ACM). Secondary outcomes comprised adverse events (AEs), hypoglycemia, and cardiovascular death.

In this study, we employed a prespecified definition of MACEs, specifically the standard 3-point MACE (MACE-3), which comprises cardiovascular death, non-fatal myocardial infarction, and non-fatal stroke. The composite renal outcomes were defined as the first occurrence of either ESRD (requiring dialysis or kidney transplantation) with a sustained decline of ≥ 40% in estimated glomerular filtration rate (eGFR) from baseline, or renal death. ACM refers to the ratio of the total number of deaths from all causes within a specific population over a given period to the total size of that population. AEs refer to any adverse, unexpected medical events occurring in patients or subjects during or after a medical intervention (such as a drug, vaccine, surgery, or medical device). This term represents the aggregate of all adverse reaction events recorded in clinical trials and safety monitoring. Hypoglycemia refers to a clinical syndrome characterized by abnormally low venous plasma glucose levels due to various causes, leading to corresponding symptoms and signs. Cardiovascular death refers to mortality primarily caused directly by cardiovascular diseases.

Studies with the characteristics below were excluded: (1) Animal or cell studies, case reports, experiment protocols, letters, editorials, reviews, or conference papers; (2) Publications with missing data or critical mistakes; (3) Duplicates; (4) Publications without available full texts.

### 2.3 Data extraction

The articles acquired from the databases were loaded into EndNote. To screen the articles, two researchers (Xiaojian Zhu and Xingjia Wang) independently assessed the titles and abstracts. Subsequently, they reviewed the full texts. Disagreements in the screening were addressed by discussion or by consulting a third researcher (Jiameng Li). Data were extracted from the included studies utilizing Excel 2016 by two researchers independently, covering first author, publication year, country or region, study number, intervention and control, treatment duration, basic characteristics of study subjects, CKD definition, and outcome measures.

### 2.4 Quality assessment

To assess the risk of bias in the incorporated studies, the Cochrane risk of bias tool version 2.0 (RoB 2.0) (9) was applied. The tool covers 5 domains: randomization, deviations from intended interventions, missing outcome data, outcome measurement, and selective reporting of the result. For each study, two researchers independently assessed the quality and rated it as “low risk”, “high risk”, or “possible risk” for each domain. Discrepancies were addressed by discussion or referred to a third researcher. A risk of bias plot was drawn to display the assessment results.

### 2.5 Statistical analysis

The outcomes, including MACEs, composite renal outcomes, ACM, AEs, hypoglycemia, and cardiovascular death, were expressed as risk ratio (RR) with 95% credible interval (CrI). Considering the heterogeneity among trials, the Bayesian hierarchical random-effects model was fitted first to make multiple comparisons of different interventions for T2DM-CKD (10, 11). R 4.2.2 and Stata 15.1 were utilized for all calculations and graphs. A Markov chain Monte Carlo (MCMC) simulation was performed based on the likelihood function and some prior assumptions. Subsequently, Bayesian inference was applied in R 4.2.2 with 500,000 iterations and 20,000 annealing times set to investigate the posterior distributions of the interrogated nodes (12-14). The node splitting method was used to evaluate local inconsistency for outcomes with a closed loop. The relationships among different interventions were presented as network diagrams. Meanwhile, comparison-adjusted funnel plots were utilized to test for potential publication bias (15, 16). Moreover, the surface under the cumulative ranking curve (SUCRA) value, ranging from 0 to 1, was utilized to rank the examined interventions. The intervention was ranked higher when the SUCRA value was higher (17, 18). League tables were generated to display the pairwise comparisons of interventions regarding each outcome.

## 3 Results

### 3.1 Literature search and screening process

**Figure 1:**
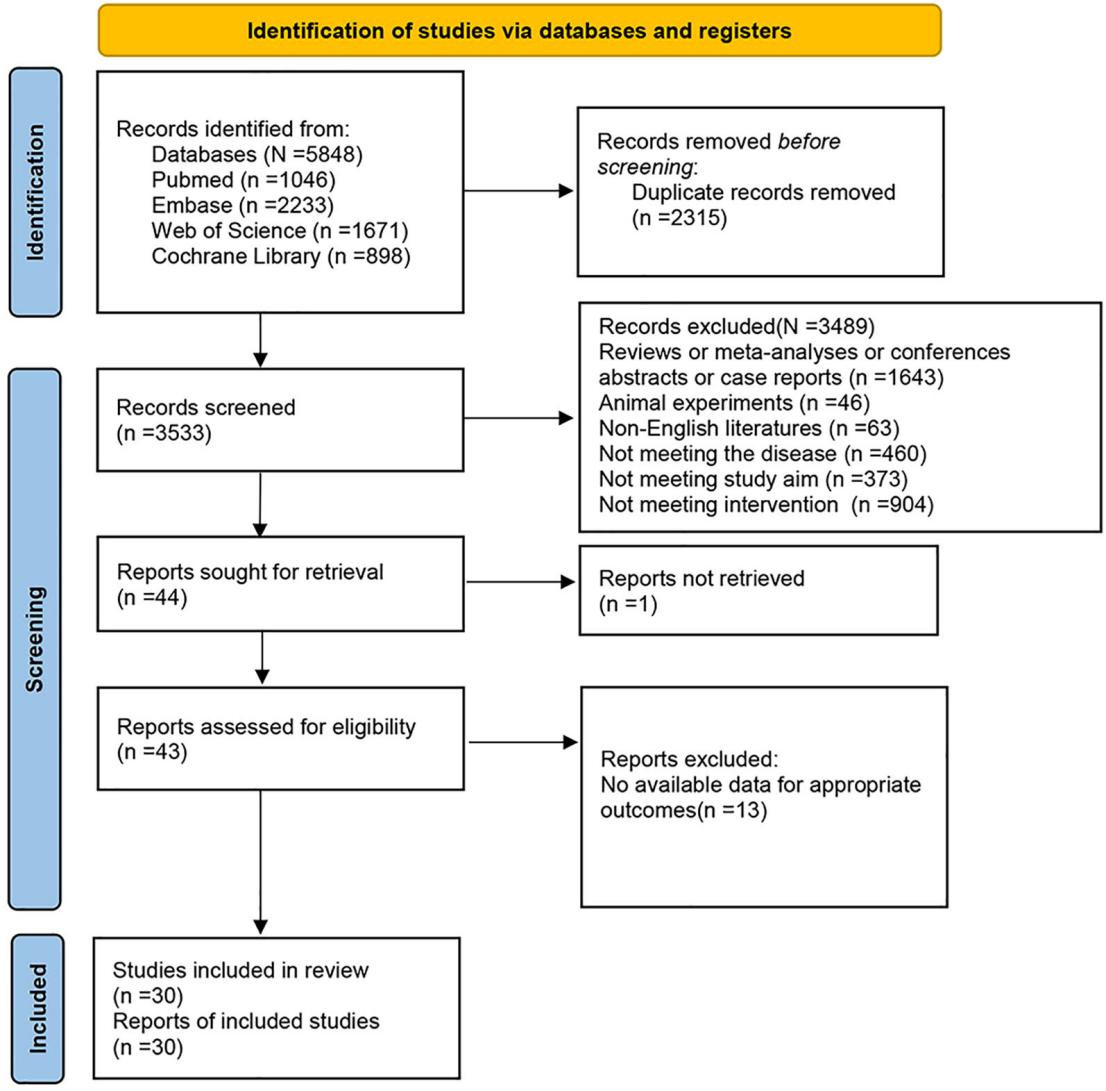
Flowchart.

In total, 5,848 articles were acquired from the initial search, with 2,315 duplicates excluded. Following the preliminary screening, 3,533 articles were discarded. Subsequently, the remaining articles were screened by full-text review with strict adherence to the above criteria. Ultimately, 30 articles were incorporated (19-48), as depicted in **Figure 1**.

### 3.2 Basic characteristics of included studies

Among the 30 incorporated studies, 7 originated from 4 countries (Denmark, the Netherlands, Japan, and India), while 23 were large-scale multicenter trials. A total of 39,844 patients were enrolled, comprising 24,970 males and 14,874 females, with ages ranging from 58.5 to 71.1 years. Among the 30 studies, 16 studies (19, 21, 22, 24, 26-29, 31, 36, 38, 44-48) compared SGLT-2is, including dapagliflozin, canagliflozin, empagliflozin, sotagliflozin, ertugliflozin, bexagliflozin, and luseogliflozin. Another 8 studies (25, 32, 35, 37, 41-44) compared GLP-1RAs, including semaglutide, exenatide, dulaglutide, cotadutide, albiglutide, and liraglutide. The remaining 10 studies (20, 23, 28, 30, 32-34, 38-40) compared DPP-4is, including saxagliptin, linagliptin, sitagliptin, trelagliptin, omarigliptin, and vildagliptin. Detailed characteristics and key findings of all included studies are presented in **Supplementary Table 2** of **Supplementary Material 1**.

### 3.3 Quality assessment results of incorporated studies

The 30 incorporated studies were examined for risk of bias, as presented in **Supplementary Figure 1** of **Supplementary Material 2**. Regarding bias arising from randomization, 3 studies (28, 29, 45) were at possible risk of bias because subjects were not randomized or treatment allocation was not concealed, while the remaining 27 studies exhibited low risk. Regarding bias due to deviations from intended interventions, 4 studies (27, 38, 42, 43) were at possible risk of bias because of no information on blinding, while the remaining 26 studies exhibited low risk. In terms of missing outcome data and outcome measurements, all studies were rated low risk of bias. Regarding bias as a result of selective reporting of the result, one study (31) was at possible risk of bias because it failed to report all pre-specified outcomes, while the remaining 29 studies were rated low risk. In summary, the overall risk of bias was low in the incorporated articles.

### 3.4 NMA results

#### 3.4.1 Network diagrams

**Figure 2:**
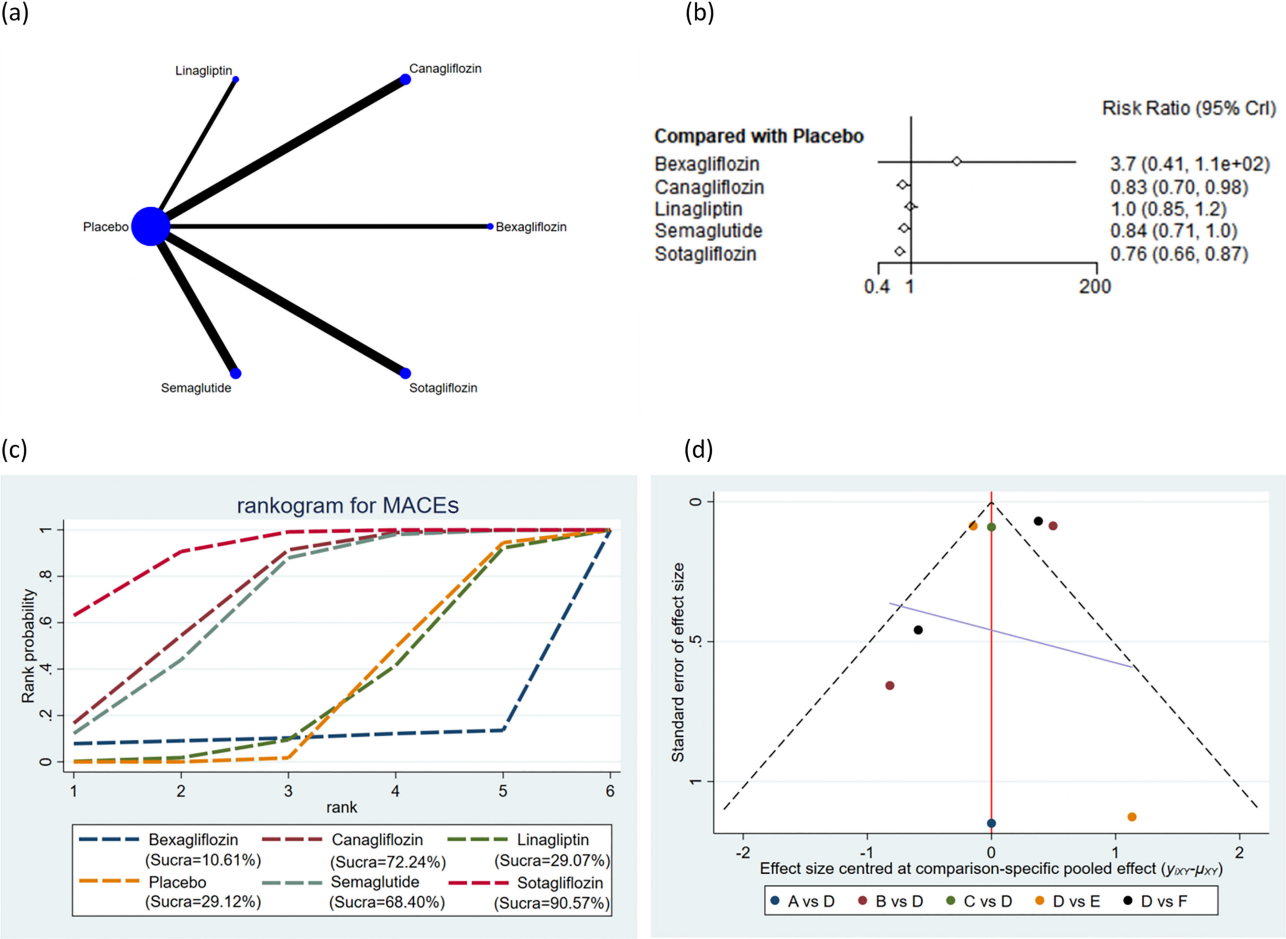
MACEs. **(a)** Network diagram; **(b)** Forest plot; **(c)** Probability line graph; **(d)** Funnel plot.

**Figure 3:**
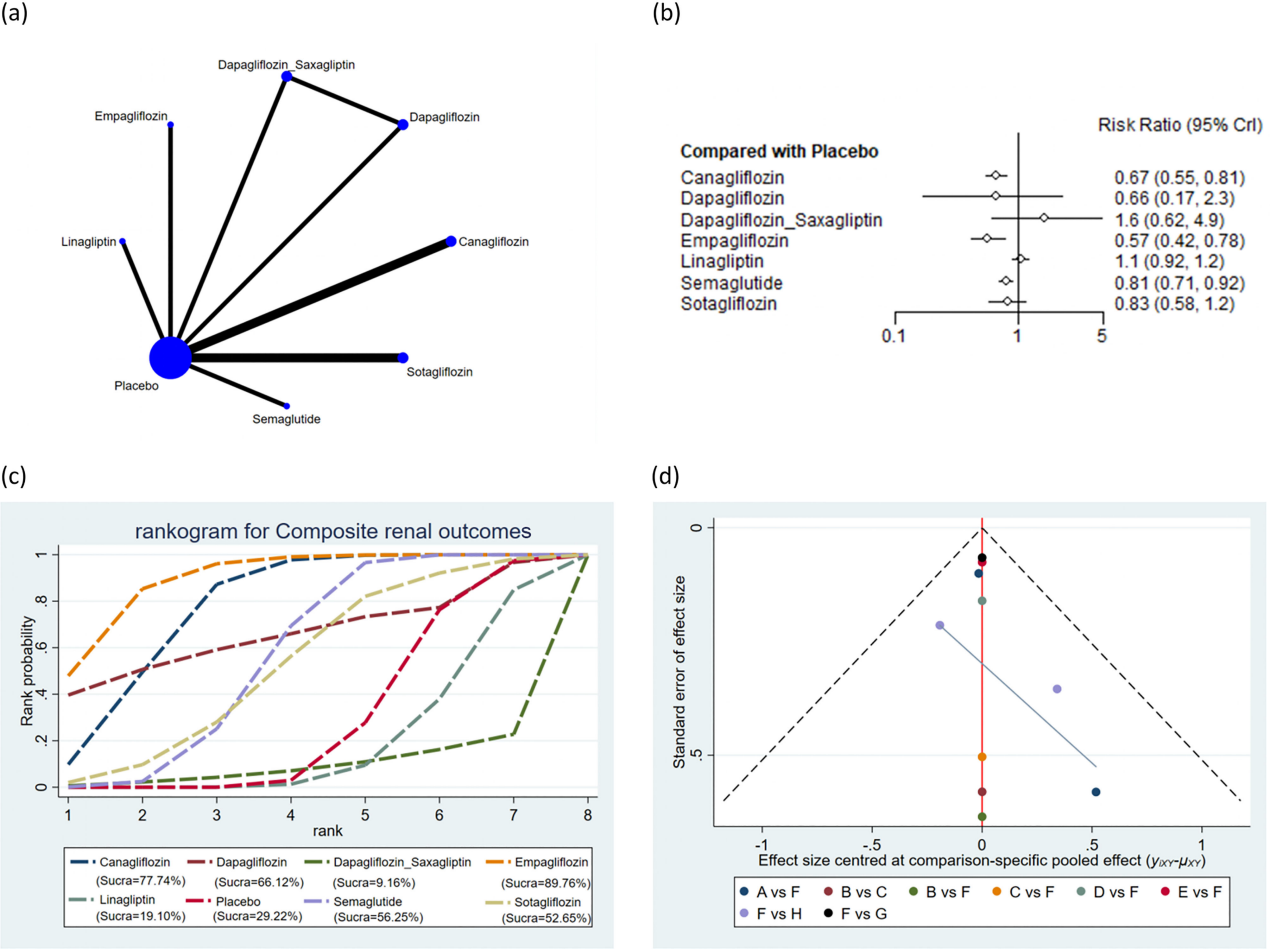
Composite renal outcomes. **(a)** Network diagram; **(b)** Forest plot; **(c)** Probability line graph; **(d)** Funnel plot.

**Figure 4:**
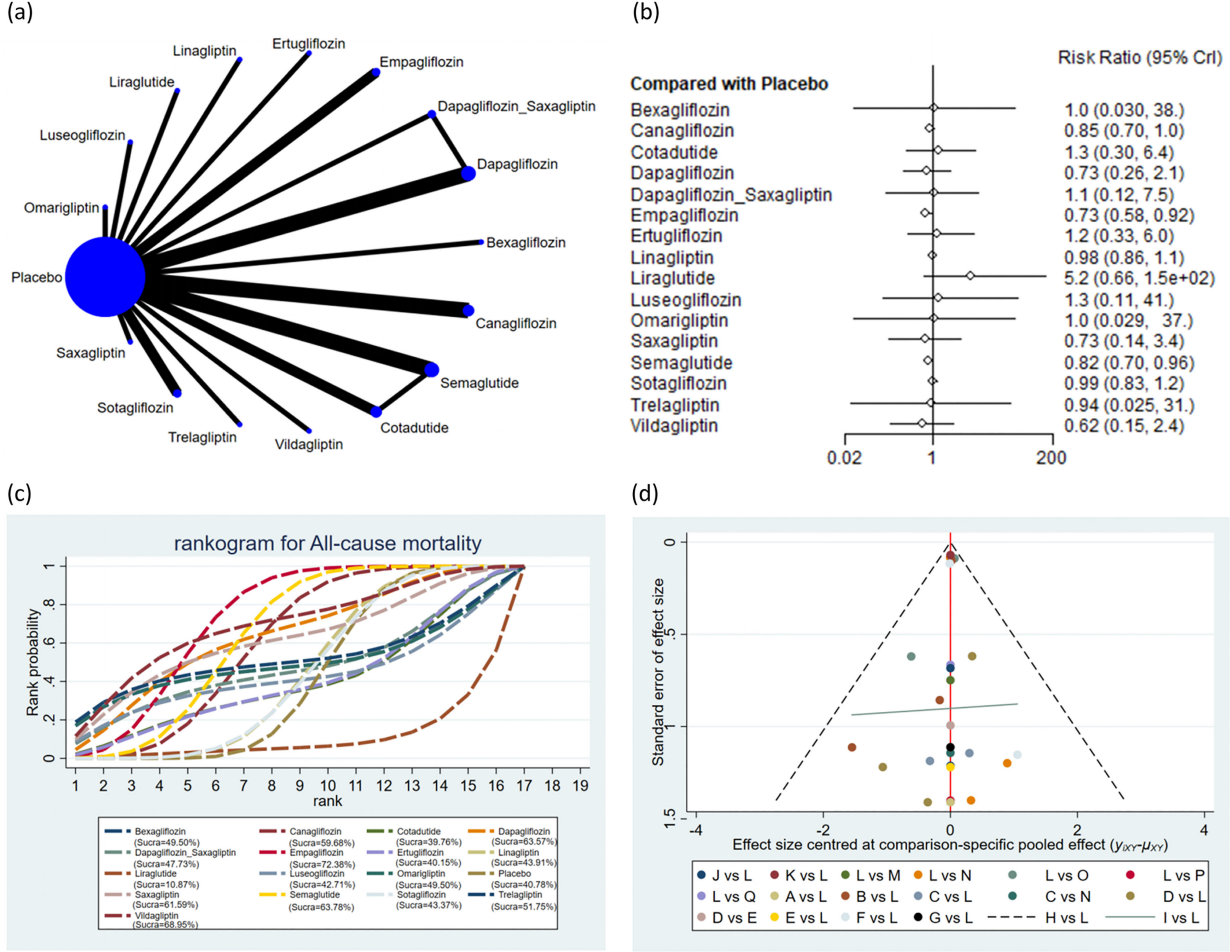
All-cause mortality. **(a)** Network diagram; **(b)** Forest plot; **(c)** Probability line graph; **(d)** Funnel plot.

**Figure 5:**
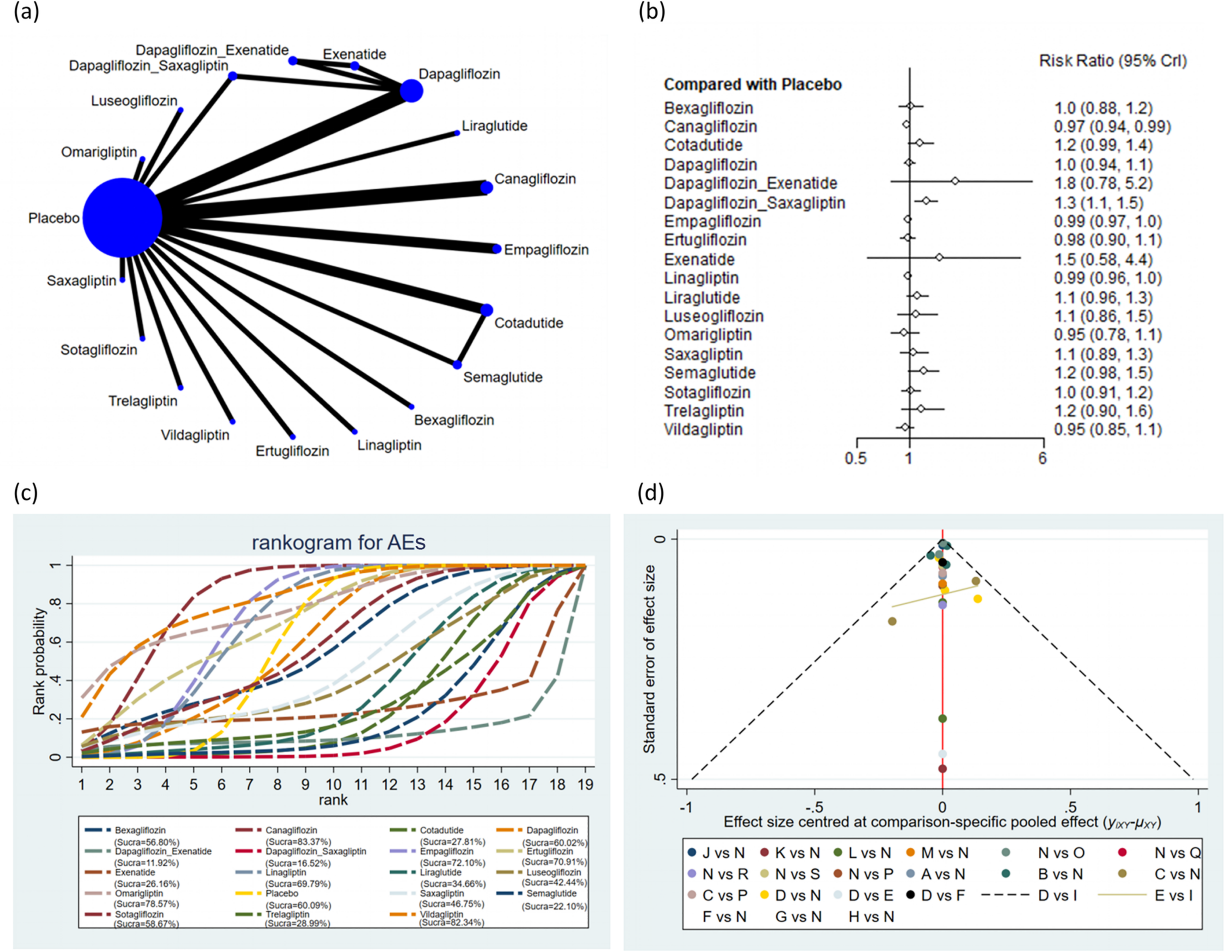
Adverse events. **(a)** Network diagram; **(b)** Forest plot; **(c)** Probability line graph; **(d)** Funnel plot.

**Figure 6:**
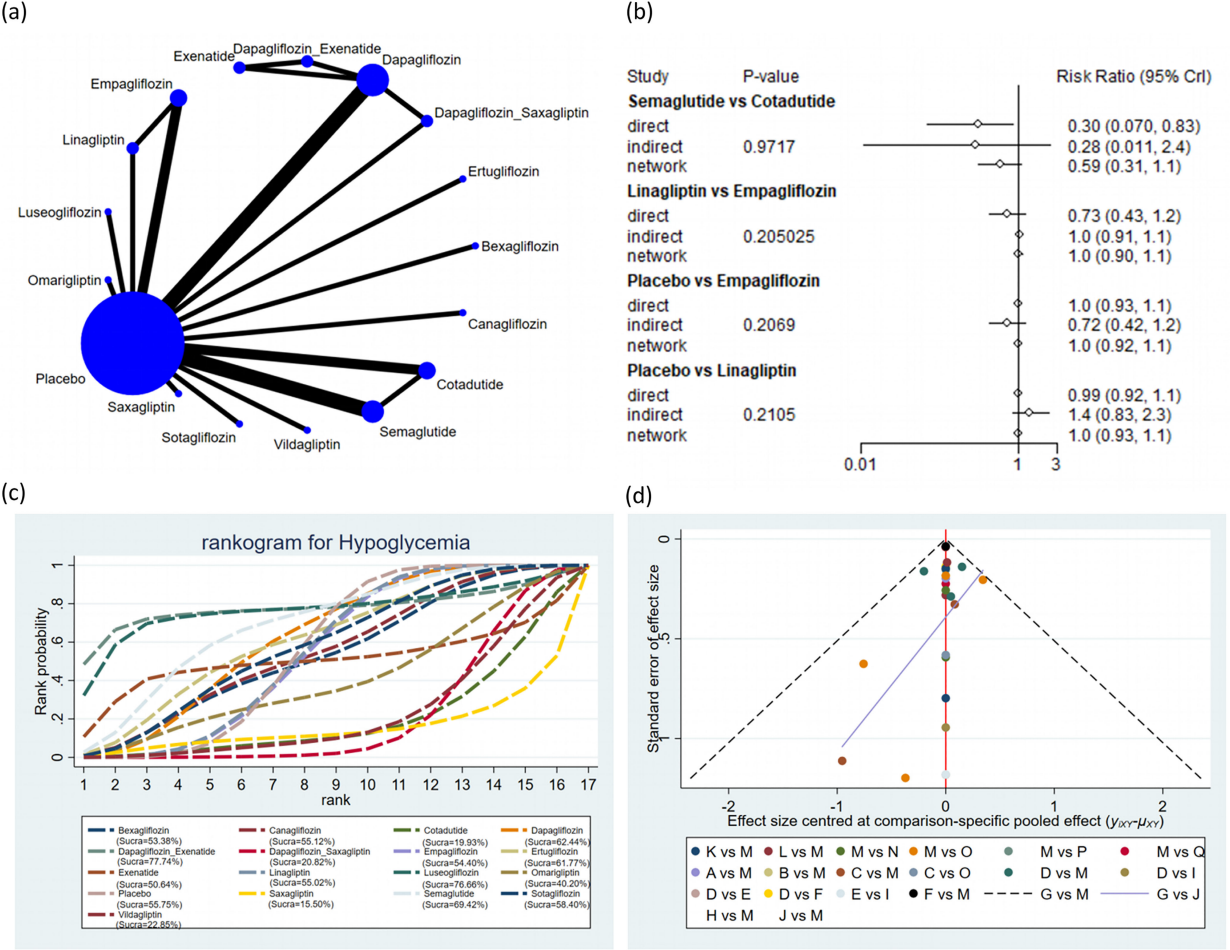
Hypoglycemia. **(a)** Network diagram; **(b)** Forest plot; **(c)** Probability line graph; **(d)** Funnel plot.

**Figure 7:**
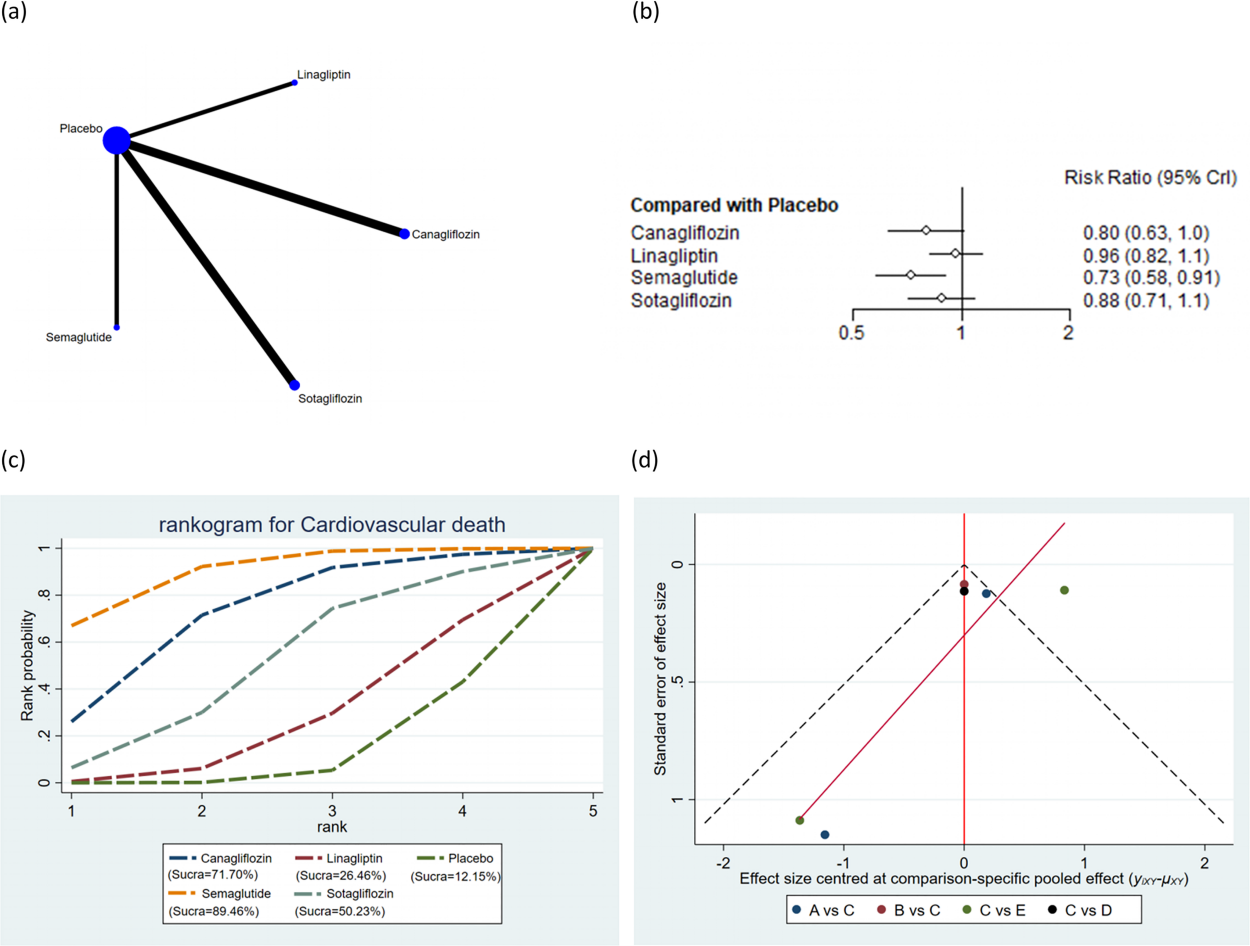
Cardiovascular death. **(a)** Network diagram; **(b)** Forest plot; **(c)** Probability line graph; **(d)** Funnel plot.

The 30 incorporated studies involved 19 distinct drug interventions: bexagliflozin, canagliflozin, cotadutide, dapagliflozin, dapagliflozin + saxagliptin, empagliflozin, ertugliflozin, linagliptin, liraglutide, luseogliflozin, omarigliptin, placebo, saxagliptin, semaglutide, sotagliflozin, trelagliptin, vildagliptin, dapagliflozin + exenatide, and exenatide. Network diagrams showing the structural associations among various interventions are presented in **Figures 2a**-**7a**. In the figures, line thickness reflects the number of articles compared pairwise, while circle diameter reflects the number of subjects treated by the intervention. Node splitting analysis of outcomes with a closed loop disclosed that all P values were > 0.05, indicating the absence of local inconsistency (**Supplementary Figures 2**, **3** of **Supplementary Material 2**).

#### 3.4.2 MACEs

A total of 8 studies encompassing 26,454 subjects reported MACEs in 2,229 subjects across 6 interventions: bexagliflozin, canagliflozin, linagliptin, placebo, semaglutide, and sotagliflozin. According to the results, the incidence of MACEs was markedly lower with canagliflozin (RR = 0.83, 95% CrI: 0.70, 0.98), semaglutide (RR = 0.84, 95% CrI: 0.71, 1.00), and sotagliflozin (RR = 0.76, 95% CrI: 0.66, 0.87) than placebo (**Figure 2b**). The incidence of MACEs was markedly lower with sotagliflozin (RR = 0.75, 95% CrI: 0.6, 0.94) than with linagliptin. The pairwise comparisons of other interventions revealed no statistically significant differences (**Supplementary Table 3** of **Supplementary Material 1**). Based on the SUCRA values, sotagliflozin (SUCRA: 90.57%), canagliflozin (SUCRA: 72.24%), and semaglutide (SUCRA: 68.40%) appeared to result in the lowest incidence of MACEs among all interventions (**Figure 2c**).

#### 3.4.3 Composite renal outcomes

A total of 8 studies encompassing 33,495 subjects reported composite renal outcomes in 2,060 subjects across 8 interventions: canagliflozin, dapagliflozin, dapagliflozin + saxagliptin, empagliflozin, linagliptin, placebo, semaglutide, and sotagliflozin. The results suggested that the incidence of composite renal outcomes was markedly lower with canagliflozin (RR = 0.67, 95% CrI: 0.55, 0.81), empagliflozin (RR = 0.57, 95% CrI: 0.42, 0.78), and semaglutide (RR = 0.81, 95% CrI: 0.71, 0.92) than placebo (**Figure 3b**). The incidence of composite renal outcomes was markedly lower with empagliflozin (RR = 0.35, 95% CrI: 0.11, 0.97) than with dapagliflozin + saxagliptin. The incidence of composite renal outcomes was markedly lower with canagliflozin (RR = 0.63, 95% CrI: 0.49, 0.80), empagliflozin (RR = 0.54, 95% CrI: 0.38, 0.76), and semaglutide (RR = 0.76, 95% CrI: 0.62, 0.92) than with linagliptin. The incidence of composite renal outcomes was markedly lower with empagliflozin (RR = 0.71, 95% CrI: 0.50, 1.00) than with semaglutide. The pairwise comparisons of other interventions revealed no statistically significant differences (**Supplementary Table 4** of **Supplementary Material 1**). Based on the SUCRA values, empagliflozin (SUCRA: 89.76%), canagliflozin (SUCRA: 77.74%), and dapagliflozin (SUCRA: 66.12%) appeared to result in the lowest incidence of composite renal outcomes among all interventions (**Figure 3c**).

#### 3.4.4 ACM

A total of 23 studies encompassing 37,676 subjects reported ACM in 2,477 subjects across 17 interventions: bexagliflozin, canagliflozin, cotadutide, dapagliflozin, dapagliflozin + saxagliptin, empagliflozin, ertugliflozin, linagliptin, liraglutide, luseogliflozin, omarigliptin, placebo, saxagliptin, semaglutide, sotagliflozin, trelagliptin, and vildagliptin. The results suggested that the ACM was markedly lower with empagliflozin (RR = 0.73, 95% CrI: 0.58, 0.92) and semaglutide (RR = 0.82, 95% CrI: 0.70, 0.96) than placebo (**Figure 4b**). The ACM was markedly lower with empagliflozin (RR = 0.74, 95% CrI: 0.57, 0.97) than with linagliptin. The ACM was markedly lower with empagliflozin (RR = 0.74, 95% CrI: 0.56, 0.98) than with sotagliflozin. The pairwise comparisons of other interventions revealed no statistically significant differences (**Supplementary Table 5** of **Supplementary Material 1**). Based on the SUCRA values, empagliflozin (SUCRA: 72.38%), vildagliptin (SUCRA: 68.95%), and semaglutide (SUCRA: 63.78%) appeared to result in the lowest incidence of ACM among all interventions (**Figure 4c**).

#### 3.4.5 AEs

A total of 21 studies encompassing 23,548 subjects reported AEs in 19,282 subjects across 19 interventions: bexagliflozin, canagliflozin, cotadutide, dapagliflozin, dapagliflozin + exenatide, dapagliflozin + saxagliptin, empagliflozin, ertugliflozin, exenatide, linagliptin, liraglutide, luseogliflozin, omarigliptin, placebo, saxagliptin, semaglutide, sotagliflozin, trelagliptin, and vildagliptin. The results suggested that the incidence of AEs was markedly lower with canagliflozin (RR = 0.97, 95% CrI: 0.94, 0.99) and empagliflozin (RR = 0.99, 95% CrI: 0.97, 1.00) than placebo (**Figure 5b**). The incidence of AEs was markedly lower with canagliflozin (RR = 0.83, 95% CrI: 0.70, 0.97), empagliflozin (RR = 0.85, 95% CrI: 0.71, 0.99), linagliptin (RR = 0.85, 95% CrI: 0.71, 1.00), and vildagliptin (RR = 0.82, 95% CrI: 0.66, 0.99) than with cotadutide. The incidence of AEs was markedly lower with canagliflozin (RR = 0.77, 95% CrI: 0.66, 0.90), dapagliflozin (RR = 0.80, 95% CrI: 0.69, 0.94), empagliflozin (RR = 0.79, 95% CrI: 0.67, 0.92), ertugliflozin (RR = 0.78, 95% CrI: 0.66, 0.94), linagliptin (RR = 0.79, 95% CrI: 0.67, 0.92), omarigliptin (RR = 0.75, 95% CrI: 0.59, 0.96), placebo (RR = 0.8, 95% CrI: 0.68, 0.93), sotagliflozin (RR = 0.81, 95% CrI: 0.67, 0.99), and vildagliptin (RR = 0.76, 95% CrI: 0.63, 0.92) than dapagliflozin + saxagliptin. The incidence of AEs was markedly lower with canagliflozin (RR = 0.80, 95% CrI: 0.65, 0.98), empagliflozin (RR = 0.81, 95% CrI: 0.66, 1.00), linagliptin (RR = 0.82, 95% CrI: 0.66, 1.00), and vildagliptin (RR = 0.79, 95% CrI: 0.62, 0.99) than with semaglutide. The pairwise comparisons of other interventions revealed no statistically significant differences (**Supplementary Table 6** of **Supplementary Material 1**). Based on the SUCRA values, canagliflozin (SUCRA: 83.37%), vildagliptin (SUCRA: 82.34%), and omarigliptin (SUCRA: 78.57%) appeared to result in the lowest incidence of AEs among all interventions (**Figure 5c**).

#### 3.4.6 Hypoglycemia

A total of 20 studies encompassing 22,196 subjects reported hypoglycemia in 5,246 subjects across 17 interventions: bexagliflozin, canagliflozin, cotadutide, dapagliflozin, dapagliflozin + exenatide, dapagliflozin + saxagliptin, empagliflozin, ertugliflozin, exenatide, linagliptin, luseogliflozin, omarigliptin, placebo, saxagliptin, semaglutide, sotagliflozin, vildagliptin. The results suggested that the incidence of hypoglycemia was markedly lower with dapagliflozin (RR = 0.71, 95% CrI: 0.55, 0.92), empagliflozin (RR = 0.75, 95% CrI: 0.58, 0.97), linagliptin (RR = 0.74, 95% CrI: 0.58, 0.97), and placebo (RR = 0.74, 95% CrI: 0.58, 0.95) than with dapagliflozin + saxagliptin (**Figure 6b**). The pairwise comparisons of other interventions revealed no statistically significant differences (**Supplementary Table 7** of **Supplementary Material 1**). Based on the SUCRA values, dapagliflozin + exenatide (SUCRA: 77.74%), luseogliflozin (SUCRA: 76.66%), and semaglutide (SUCRA: 69.42%) appeared to result in the lowest incidence of hypoglycemia among all interventions (**Figure 6c**).

#### 3.4.7 Cardiovascular death

A total of 6 studies encompassing 26,082 subjects reported cardiovascular death in 1,396 subjects across 5 interventions: canagliflozin, linagliptin, placebo, semaglutide, and sotagliflozin. The results suggested that the incidence of cardiovascular death was markedly lower with semaglutide (RR = 0.73, 95% CrI: 0.58, 0.91) than placebo (**Figure 7b**). The incidence of cardiovascular death was markedly lower with semaglutide (RR = 0.75, 95% CrI: 0.57, 1.00) than with linagliptin. The pairwise comparisons of other interventions revealed no statistically significant differences (**Supplementary Table 8** of **Supplementary Material 1**). Based on the SUCRA values, semaglutide (SUCRA: 89.46%), canagliflozin (SUCRA: 71.70%), and sotagliflozin (SUCRA: 50.23%) appeared to result in the lowest incidence of cardiovascular death among all interventions (**Figure 7c**).

### 3.5 Subgroup analyses

#### 3.5.1 ACM

Subgroup analysis by treatment duration revealed that for treatment duration ≤ 26 weeks, vildagliptin (SUCRA: 72.73%), canagliflozin (SUCRA: 68.80%), and placebo (SUCRA: 59.36%) were ranked as the three potentially most effective interventions for reducing ACM. For treatment duration > 26 weeks, dapagliflozin (SUCRA: 79.07%), empagliflozin (SUCRA: 76.86%), and semaglutide (SUCRA: 64.42%) were ranked as the three potentially optimal interventions for reducing ACM (**Supplementary Figures 4**, **5** of **Supplementary Material 2** and **Supplementary Tables 9**, **10** of **Supplementary Material 1**).

#### 3.5.2 AEs

Subgroup analysis by treatment duration revealed that for treatment duration ≤ 26 weeks, vildagliptin (SUCRA: 80.77%), dapagliflozin (SUCRA: 80.67%), and omarigliptin (SUCRA: 78.71%) were ranked as the three potentially most effective interventions for reducing AEs. For treatment duration > 26 weeks, canagliflozin (SUCRA: 86.19%), empagliflozin (SUCRA: 63.63%), and ertugliflozin (SUCRA: 61.23%) were ranked as the three potentially most effective interventions for reducing AEs (**Supplementary Figures 6**, **7** of **Supplementary Material 2** and **Supplementary Tables 11**, **12** of **Supplementary Material 1**).

#### 3.5.3 Hypoglycemia

Subgroup analysis by treatment duration revealed that for treatment duration ≤ 26 weeks, semaglutide (SUCRA: 89.91%), luseogliflozin (SUCRA: 73.13%), and dapagliflozin + exenatide (SUCRA: 71.25%) were ranked as the three potentially most effective interventions for alleviating hypoglycemia. For treatment duration > 26 weeks, dapagliflozin (SUCRA: 83.98%), ertugliflozin (SUCRA: 59.53%), and sotagliflozin (SUCRA: 54.27%) were ranked as the three potentially most effective interventions for alleviating hypoglycemia (**Supplementary Figures 8**, **9** of **Supplementary Material 2** and **Supplementary Tables 13**, **14** of **Supplementary Material 1**).

### 3.6 Publication bias

Publication bias across all outcomes was assessed utilizing funnel plots and Egger’s test. Symmetric funnel plots for all outcomes suggested no publication bias (**Figures 2d**-**7d**). Egger’s test indicated no publication bias in terms of MACEs (p = 0.535), composite renal outcomes (p = 0.288), ACM (p = 0.539), AEs (p = 0.667), hypoglycemia (p = 0.489), and cardiovascular death (p = 0.303).

## 4 Discussion

This is the first NMA comparing specific novel antidiabetic drugs for efficacy and safety in populations with T2DM-CKD. The latest data from 30 eligible RCTs were analyzed. According to our NMA, sotagliflozin demonstrated the greatest probability of being the optimal intervention for reducing MACEs (SUCRA: 90.57%), showing significant superiority over placebo and linagliptin. Empagliflozin exhibited the greatest probability of being the most effective in improving composite renal outcomes (SUCRA: 89.76%), markedly outperforming placebo, dapagliflozin + saxagliptin, linagliptin, and semaglutide. Empagliflozin was most likely to be the most effective in reducing ACM (SUCRA: 72.38%), with substantial superiority over placebo, linagliptin, and sotagliflozin. Canagliflozin demonstrated the highest likelihood of being the optimal intervention for reducing AEs (SUCRA: 83.37%), significantly outperforming placebo, cotadutide, dapagliflozin + saxagliptin, and semaglutide. Dapagliflozin + exenatide represented the most likely optimal intervention for reducing hypoglycemic events (SUCRA: 77.74%), with substantially better effectiveness than placebo. Semaglutide exhibited the highest probability of being the most effective in reducing cardiovascular mortality (SUCRA: 89.46%), showing considerable superiority over placebo and linagliptin.

This study revealed that compared to placebo and other drugs, sotagliflozin significantly reduced the incidence of MACEs. This finding further supports the conclusion of a previous meta-analysis that SGLT-2is exhibit significant efficacy in lowering the risk of MACEs and represent one of the effective treatment options available at present (7). The SCORED trial (22) and SOTA-CKD4 trial (24) have validated the cardioprotective effects of sotagliflozin across various patient populations (CKD and severe renal insufficiency). The SCORED trial has demonstrated in a large population that sotagliflozin markedly reduces the risk of composite endpoints involving heart failure, while the SOTA-CKD4 trial has shown improved cardiovascular safety in high-risk subgroups. This strongly supports the recommendations in the latest clinical guidelines from the Kidney Disease: Improving Global Outcomes (KDIGO) organization (49) and the American Diabetes Association (ADA)/European Association for the Study of Diabetes (EASD) (50). These guidelines emphasize that SGLT-2is should be used in populations with T2DM-CKD to lower the incidence of MACEs.

Moreover, empagliflozin significantly reduces the incidence of composite renal outcomes. This further supports the conclusion of prior meta-analyses that SGLT-2is substantially lower the risk of kidney-specific composite endpoints (6, 7). The EMPA-REG OUTCOME trial (46) has further confirmed the renal protective effects of empagliflozin in high-risk T2DM patients, with a 39% decline in the risk of adverse composite renal outcomes in the empagliflozin group (hazard ratio 0.61; 95% CrI 0.53-0.70; P < 0.001). The ADA/KDIGO (51) consensus report has emphasized that the benefits of empagliflozin for renal outcomes are independent of its glucose-lowering effects, primarily through improved glomerular hemodynamics, reduced inflammation, and decreased fibrosis.

Empagliflozin significantly reduces the ACM. A previous meta-analysis (6) has reported that either SGLT-2is or GLP-1RAs lower the ACM by 20%. Our study further demonstrated that empagliflozin lowered the incidence of ACM by 27%, showing a marked effect. The EMPA-REG RENAL trial (21) and EMPA-REG OUTCOME trial (46) have demonstrated robust benefits to renal and survival outcomes in populations with T2DM-CKD. The ADA/KDIGO (51) consensus report has recommended SGLT-2is (e.g., empagliflozin) for populations with T2DM-CKD (eGFR ≥ 20 mL/min/1.73 m²) to lower the cardiovascular events rate and mortality rate, irrespective of proteinuria. Meanwhile, subgroup analyses indicated that vildagliptin was the most effective when treatment duration was ≤ 26 weeks, while dapagliflozin showed the best efficacy when treatment duration was > 26 weeks. This suggested that treatment effects may change over time. This highlights the need for individualized selection of antidiabetic drugs based on disease stage, though SGLT-2is remain the preferred choice overall.

Compared to placebo and other drugs, canagliflozin markedly reduces the incidence of AEs. Dapagliflozin + exenatide markedly reduces the incidence of hypoglycemia. Semaglutide markedly reduces the incidence of cardiovascular death. SGLT-2is inhibit the reabsorption of sodium and glucose by the proximal renal tubule, thus mitigating glomerular hyperfiltration and improving hemodynamics. Consequently, they reduce inflammation and oxidative stress, alleviating renal burden and lowering the risk of cardiovascular events (52). The non-insulin-dependent glucose-lowering mechanism of SGLT-2is also leads to a significant reduction of hypoglycemia-related adverse events (53), thereby lowering the incidence of adverse events. GLP-1RAs directly protect the cardiovascular system by inhibiting the release of inflammatory cytokines and mitigating oxidative damage (54). They indirectly reduce the risk of cardiovascular events by improving hemodynamics through vasodilation and thereby lowering blood pressure and cardiac load (55). They also promote weight loss and glycemic control, reducing the burden of cardiovascular disease (56), thereby lowering the incidence of cardiovascular death. The DECADE trial (44) has demonstrated that the SGLT-2i + GLP-1RA therapy efficiently lowers blood glucose and markedly reduces the risk of hypoglycemia through the synergistic effects of multiple mechanisms. These mechanisms include non-insulin-dependent glucose-lowering pathways, glucose-dependent insulin regulation, and improved renal hemodynamics and overall metabolic environment.

Subgroup analysis of adverse events revealed that with treatment duration ≤ 26 weeks, vildagliptin exhibited the greatest reduction in the incidence of AEs, while semaglutide performed the best in lowering the incidence of hypoglycemia. With treatment duration > 26 weeks, canagliflozin exhibited the greatest reduction in the incidence of AEs, while dapagliflozin performed the best in lowering the incidence of hypoglycemia. Treatment duration significantly influenced the safety performance of different antidiabetic agents. This may be largely attributable to the mechanism of action of the agents, the metabolic adaptation in patients, and the cumulative effects during long-term therapy (57). In short-term treatment (≤ 26 weeks), vildagliptin performed the best in reducing the overall incidence of AEs. This is primarily because of its glucose-dependent insulinotropic properties as a DPP-4i, which significantly reduce the risk of hypoglycemia. Meanwhile, its anti-inflammatory properties may also indirectly improve gastrointestinal tolerance, thus lowering the incidence of drug-related AEs in the short term (57). Semaglutide performed the best in reducing the risk of hypoglycemia in the short term, which may be attributed to its glucose-dependent hypoglycemic mechanism as a GLP-1RA. This avoids unnecessary insulin secretion and minimizes hypoglycemic events. In long-term treatment (> 26 weeks), canagliflozin performed the best in reducing the overall incidence of AEs. This may result from its cumulative long-term metabolic benefits (e.g., sustained weight loss, improved blood pressure) as an SGLT-2i. Network meta-analyses have indicated that canagliflozin significantly reduces hospitalization for heart failure and kidney function progression, possibly representing improved overall safety (58, 59). Dapagliflozin performs the best in controlling the risk of hypoglycemia during long-term treatment, which is related to its insulin-independent hypoglycemic pathway. It can effectively manage hypoglycemia even when administered with insulin or sulfonylureas.

In summary, the results of this NMA provide profound practical guidance for the clinical management of T2DM-CKD. First, it enhances precision in treatment strategies. By clarifying the ranking of different novel antidiabetic drugs in terms of key outcomes, clinicians can make stratified decisions based on individual patient characteristics, thereby developing personalized treatment strategies. For instance, sotagliflozin may be preferred for populations with high risk for MACEs, while empagliflozin may be favored for patients with rapidly deteriorating kidney function. Second, these findings fill the gap in existing guidelines by promoting outcome-oriented drug selection instead of recommending specific drugs. This approach optimizes the balance between efficacy and safety in complex clinical scenarios, such as the combined use of multiple drugs or fluctuations in disease progression. Moreover, subgroup analyses reveal that the effects of different treatment periods may vary, for instance, the short-term safety of DPP-4is and the long-term cumulative benefits of SGLT-2is. This underscores the importance of dynamic monitoring and regimen adjustments, shifting clinical practice from static protocols toward adaptive management. Ultimately, this evidence-driven individualized pathway reduces the overall burden of complications and improves treatment compliance while optimizing the allocation of healthcare resources. It provides a feasible framework for long-term management of T2DM-CKD.

### 4.1 Limitations

This study has several limitations. First, significant clinical heterogeneity exists in the definitions and staging of CKD across the included trials (ranging from stage 1 to ESRD requiring dialysis), and the evidence networks for some outcomes (e.g., MACEs and cardiovascular death) are sparse due to the limited number of available studies, which reduces the certainty of treatment rankings. Second, although standardized definitions for MACEs and composite renal outcomes were applied in this analysis, heterogeneity exists in the definitions of these endpoints across the included trials (e.g., variations in component events or eGFR decline thresholds), which should be considered when interpreting the pooled estimates. Third, although subgroup analyses by follow-up duration are implemented, some subgroups have small sample sizes and lack comparisons among multiple interventions. This potentially leads to negative results in certain interventions and placebo. Furthermore, except for ACM and hypoglycemia, data from direct comparisons for specific novel antidiabetic drugs are lacking. Hence, it is impossible to assess the consistency between indirect and direct comparisons. Besides, some studies have a possible risk of bias from randomization or blinding. These methodological flaws may compromise the reliability and generalizability of the overall evidence. Finally, individual patient data are lacking. Hence, adjustment for confounding factors such as baseline eGFR and HbA1c levels, albuminuria, or background therapies is infeasible.

## 5 Conclusion

This NMA of 30 RCTs provided comparative efficacy and safety profiles of novel antidiabetic drugs in patients with T2DM and comorbid CKD. Our findings indicated that, compared with other drugs, sotagliflozin was most likely to be the most effective in reducing MACEs. Empagliflozin was most likely to exhibit the best efficacy in improving composite renal outcomes and reducing ACM. Canagliflozin was most likely to have the greatest efficacy in reducing AEs. Dapagliflozin + exenatide was most likely to represent the best intervention for reducing hypoglycemic events. Semaglutide was most likely to perform the best in lowering cardiovascular mortality. These findings suggest that the choice of the optimal antidiabetic drug for T2DM-CKD should be individualized based on the predominant risk in the patient (e.g., cardiovascular disease, renal disease, or hypoglycemia) and the specific treatment goal.

## Data Availability

The original contributions presented in the study are included in the article/**Supplementary Material**. Further inquiries can be directed to the corresponding author.

## Supplementary material

Supplementary Table 1 Search strategy.

Supplementary Table 2 Baseline and outcome.

Supplementary Table 3 Comparison of MACEs in patients with type 2 diabetes and chronic kidney disease.

Supplementary Table 4 Comparison of composite renal outcomes in patients with type 2 diabetes and chronic kidney disease.

Supplementary Table 5 Comparison of all-cause mortality in patients with type 2 diabetes and chronic kidney disease.

Supplementary Table 6 Comparison of AEs in patients with type 2 diabetes and chronic kidney disease.

Supplementary Table 7 Comparison of hypoglycemia in patients with type 2 diabetes and chronic kidney disease.

Supplementary Table 8 Comparison of cardiovascular death in patients with type 2 diabetes and chronic kidney disease.

Supplementary Table 9 Comparison of all-cause mortality(≤26weeks) in patients with type 2 diabetes and chronic kidney disease.

Supplementary Table 10 Comparison of all-cause mortality(>26weeks) in patients with type 2 diabetes and chronic kidney disease.

Supplementary Table 11 Comparison of AEs (≤26weeks) in patients with type 2 diabetes and chronic kidney disease.

Supplementary Table 12 Comparison of AEs (>26weeks) in patients with type 2 diabetes and chronic kidney disease.

Supplementary Table 13 Comparison of hypoglycemia (≤26weeks) in patients with type 2 diabetes and chronic kidney disease.

Supplementary Table 14 Comparison of hypoglycemia (>26weeks) in patients with type 2 diabetes and chronic kidney disease.

Supplementary Figure 1 Risk of bias assessment graph.

Supplementary Figure 2 Node-splitting analysis for all-cause mortality.

Supplementary Figure 3 Node-splitting analysis for hypoglycemia.

Supplementary Figure 4 All-cause mortality(≤26weeks).

Supplementary Figure 5 All-cause mortality(>26weeks).

Supplementary Figure 6 AEs (≤26weeks).

Supplementary Figure 7 AEs (>26weeks).

Supplementary Figure 8 Hypoglycemia(≤26weeks).

Supplementary Figure 9 Hypoglycemia(>26weeks).
