# Supplementary material for: Efficacy and safety of novel antidiabetic drugs in patients with type 2 diabetes and chronic kidney disease: a network meta-analysis
Source: Front Endocrinol (Lausanne). 2026 Mar 31;17:1750615. doi: 10.3389/fendo.2026.1750615 (PMC13076170; doi:10.3389/fendo.2026.1750615)
Supplement: Supplementary file 1 [file DataSheet2.docx]

**Table S1** Search strategy

**Embase**

| #1 | 'adult onset diabetes':ti,ab,kw OR 'diabetes mellitus type 2':ti,ab,kw OR 'diabetes mellitus type ii':ti,ab,kw OR 'diabetes type 2':ti,ab,kw OR 'diabetes type ii':ti,ab,kw OR 'insulin independent diabetes':ti,ab,kw OR 'ketosis resistant diabetes mellitus':ti,ab,kw OR 'maturity onset diabetes':ti,ab,kw OR 'mody':ti,ab,kw OR 'niddm':ti,ab,kw OR 'non insulin dependent diabetes':ti,ab,kw OR 'noninsulin dependent diabetes':ti,ab,kw OR 'slow onset diabetes mellitus':ti,ab,kw OR 'stable diabetes mellitus':ti,ab,kw OR 't2dm':ti,ab,kw OR 'tiidm':ti,ab,kw OR 'type 2 diabetes':ti,ab,kw OR 'type ii diabetes':ti,ab,kw |
| --- | --- |
| #2 | 'non insulin dependent diabetes mellitus'/exp |
| #3 | 'chronic kidney disease*':ti,ab,kw OR 'chronic kidney disorder':ti,ab,kw OR 'chronic kidney failure':ti,ab,kw OR 'chronic kidney insufficienc*':ti,ab,kw OR 'chronic nephropathy':ti,ab,kw OR 'chronic renal disease*':ti,ab,kw OR 'chronic renal failure':ti,ab,kw OR 'chronic renal insufficienc*':ti,ab,kw OR 'kidney chronic failure':ti,ab,kw |
| #4 | 'chronic kidney failure'/exp |
| #5 | 'gliflozin*':ti,ab,kw OR 'sodium dependent glucose cotransporter 2 inhibitor':ti,ab,kw OR 'sodium glucose cotransporter 2 inhibitor':ti,ab,kw OR 'sodium glucose co-transporter 2 inhibitor':ti,ab,kw OR 'sodium glucose transporter 2 inhibitor*':ti,ab,kw OR 'sglt2':ti,ab,kw OR 'sglt 2':ti,ab,kw OR 'canagliflozin':ti,ab,kw OR 'dapagliflozin':ti,ab,kw OR 'empagliflozin':ti,ab,kw OR 'ipragliflozin':ti,ab,kw OR 'tofogliflozin':ti,ab,kw OR 'luseogliflozin':ti,ab,kw OR 'remogliflozin':ti,ab,kw OR 'ertugliflozin':ti,ab,kw OR 'sotagliflozin':ti,ab,kw OR 'bexagliflozin':ti,ab,kw |
| #6 | 'sodium glucose cotransporter 2 inhibitor'/exp |
| #7 | 'dipeptidyl peptidase 4 inhibitor*':ti,ab,kw OR 'dipeptidyl peptidase iv inhibitor*':ti,ab,kw OR 'dipeptidylpeptidase 4 inhibitor':ti,ab,kw OR 'dipeptidylpeptidase iv inhibitor':ti,ab,kw OR 'gliptin*':ti,ab,kw OR 'dpp4':ti,ab,kw OR 'dpp 4':ti,ab,kw OR 'dpp iv':ti,ab,kw OR 'alogliptin':ti,ab,kw OR 'anagliptin':ti,ab,kw OR 'gemigliptin':ti,ab,kw OR 'linagliptin':ti,ab,kw OR 'saxagliptin':ti,ab,kw OR 'sitagliptin':ti,ab,kw OR 'teneligliptin':ti,ab,kw OR 'vildagliptin':ti,ab,kw |
| #8 | 'dipeptidyl peptidase iv inhibitor'/exp |
| #9 | 'glp 1':ti,ab,kw OR 'glucagon like peptide 1':ti,ab,kw OR 'glucagon like peptide i':ti,ab,kw OR 'insulinotropin':ti,ab,kw OR 'efpeglenatide':ti,ab,kw OR 'dulaglutide':ti,ab,kw OR 'semaglutide':ti,ab,kw OR 'taspoglutide':ti,ab,kw OR 'albiglutide':ti,ab,kw OR 'tirzepatide':ti,ab,kw OR 'liraglutide':ti,ab,kw OR 'lixisenatide':ti,ab,kw OR 'exenatide':ti,ab,kw |
| #10 | 'glucagon like peptide 1'/exp |
| #11 | 'randomized controlled trials as topic':ti,ab,kw OR 'random*':ti,ab,kw OR 'placebo':ti,ab,kw OR 'control*':ti,ab,kw OR 'randomized controlled trial':ti,ab,kw |
| #12 | 'randomized controlled trial'/exp |
| #13 | (#1 OR #2) AND (#3 OR #4) AND (#5 OR #6 OR #7 OR #8 OR #9 OR #10) AND (#11 OR #12) |

**Pubmed**

| Search number | Query |
| --- | --- |
| 1 | "adult onset diabetes"[Title/Abstract] OR "diabetes mellitus type 2"[Title/Abstract] OR "diabetes mellitus type ii"[Title/Abstract] OR "diabetes type 2"[Title/Abstract] OR "diabetes type II"[Title/Abstract] OR "insulin independent diabetes"[Title/Abstract] OR "ketosis resistant diabetes mellitus"[Title/Abstract] OR "Maturity Onset Diabetes"[Title/Abstract] OR "MODY"[Title/Abstract] OR "NIDDM"[Title/Abstract] OR "non insulin dependent diabetes"[Title/Abstract] OR "noninsulin dependent diabetes"[Title/Abstract] OR "Slow Onset Diabetes Mellitus"[Title/Abstract] OR "Stable Diabetes Mellitus"[Title/Abstract] OR "T2DM"[Title/Abstract] OR "TIIDM"[Title/Abstract] OR "type 2 diabetes"[Title/Abstract] OR "type II diabetes"[Title/Abstract] |
| 2 | Diabetes Mellitus, Type 2[MeSH Terms] |
| 3 | "Chronic Kidney Disease*"[Title/Abstract] OR "chronic kidney disorder"[Title/Abstract] OR "chronic kidney failure"[Title/Abstract] OR "Chronic Kidney Insufficienc*"[Title/Abstract] OR "chronic nephropathy"[Title/Abstract] OR "Chronic Renal Disease*"[Title/Abstract] OR "chronic renal failure"[Title/Abstract] OR "Chronic Renal Insufficienc*"[Title/Abstract] OR "kidney chronic failure"[Title/Abstract] |
| 4 | Renal Insufficiency, Chronic[MeSH Terms] |
| 5 | "Gliflozin*"[Title/Abstract] OR "sodium dependent glucose cotransporter 2 inhibitor"[Title/Abstract] OR "sodium glucose cotransporter 2 inhibitor"[Title/Abstract] OR "sodium glucose co-transporter 2 inhibitor"[Title/Abstract] OR "Sodium Glucose Transporter 2 Inhibitor*"[Title/Abstract] OR "SGLT2"[Title/Abstract] OR "SGLT 2"[Title/Abstract] OR "canagliflozin"[Title/Abstract] OR "dapagliflozin"[Title/Abstract] OR "Empagliflozin"[Title/Abstract] OR "ipragliflozin"[Title/Abstract] OR "tofogliflozin"[Title/Abstract] OR "luseogliflozin"[Title/Abstract] OR "remogliflozin"[Title/Abstract] OR "ertugliflozin"[Title/Abstract] OR "sotagliflozin"[Title/Abstract] OR "bexagliflozin"[Title/Abstract] |
| 6 | Sodium-Glucose Transporter 2 Inhibitors[MeSH Terms] |
| 7 | "Dipeptidyl Peptidase 4 Inhibitor*"[Title/Abstract] OR "Dipeptidyl Peptidase IV Inhibitor*"[Title/Abstract] OR "dipeptidylpeptidase 4 inhibitor"[Title/Abstract] OR "dipeptidylpeptidase IV inhibitor"[Title/Abstract] OR "Gliptin*"[Title/Abstract] OR "DPP4"[Title/Abstract] OR "DPP 4"[Title/Abstract] OR "DPP IV"[Title/Abstract] OR "Alogliptin"[Title/Abstract] OR "Anagliptin"[Title/Abstract] OR "Gemigliptin"[Title/Abstract] OR "Linagliptin"[Title/Abstract] OR "Saxagliptin"[Title/Abstract] OR "Sitagliptin"[Title/Abstract] OR "Teneligliptin"[Title/Abstract] OR "Vildagliptin"[Title/Abstract] |
| 8 | Dipeptidyl-Peptidase IV Inhibitors[MeSH Terms] |
| 9 | "GLP 1"[Title/Abstract] OR "Glucagon Like Peptide 1"[Title/Abstract] OR "glucagon like peptide i"[Title/Abstract] OR "insulinotropin"[Title/Abstract] OR "Efpeglenatide"[Title/Abstract] OR "Dulaglutide"[Title/Abstract] OR "Semaglutide"[Title/Abstract] OR "Taspoglutide"[Title/Abstract] OR "Albiglutide"[Title/Abstract] OR "tirzepatide"[Title/Abstract] OR "Liraglutide"[Title/Abstract] OR "Lixisenatide"[Title/Abstract] OR "Exenatide"[Title/Abstract] |
| 10 | Glucagon-Like Peptide 1[MeSH Terms] |
| 11 | "Randomized Controlled Trials as Topic"[Title/Abstract] OR "random*"[Title/Abstract] OR "placebo"[Title/Abstract] OR "control*"[Title/Abstract] OR "randomized controlled trial "[Title/Abstract] |
| 12 | Randomized Controlled Trials as Topic[MeSH Terms] |
| 13 | (#1 OR #2) AND (#3 OR #4) AND (#5 OR #6 OR #7 OR #8 OR #9 OR #10) AND (#11 OR #12) |

**Web**

| #1 | TS=(“adult onset diabetes" OR "diabetes mellitus type 2" OR "diabetes mellitus type ii" OR "diabetes type 2" OR "diabetes type II" OR "insulin independent diabetes" OR "ketosis resistant diabetes mellitus" OR "Maturity Onset Diabetes" OR "MODY" OR "NIDDM" OR "non insulin dependent diabetes" OR "noninsulin dependent diabetes" OR "Slow Onset Diabetes Mellitus" OR "Stable Diabetes Mellitus" OR "T2DM" OR "TIIDM" OR "type 2 diabetes" OR "type II diabetes") and Preprint Citation Index (Exclude – Database) |
| --- | --- |
| #2 | TS=(“Chronic Kidney Disease*" OR "chronic kidney disorder" OR "chronic kidney failure" OR "Chronic Kidney Insufficienc*" OR "chronic nephropathy" OR "Chronic Renal Disease*" OR "chronic renal failure" OR "Chronic Renal Insufficienc*" OR "kidney chronic failure" ) and Preprint Citation Index (Exclude – Database) |
| #3 | TS=(“Gliflozin*" OR "sodium dependent glucose cotransporter 2 inhibitor" OR "sodium glucose cotransporter 2 inhibitor" OR "sodium glucose co-transporter 2 inhibitor" OR "Sodium Glucose Transporter 2 Inhibitor*" OR "SGLT2" OR "SGLT 2" OR "canagliflozin" OR "dapagliflozin" OR "Empagliflozin" OR "ipragliflozin" OR "tofogliflozin" OR "luseogliflozin" OR "remogliflozin" OR "ertugliflozin" OR "sotagliflozin" OR "bexagliflozin") and Preprint Citation Index (Exclude – Database) |
| #4 | TS=(“Dipeptidyl Peptidase 4 Inhibitor*" OR "Dipeptidyl Peptidase IV Inhibitor*" OR "dipeptidylpeptidase 4 inhibitor" OR "dipeptidylpeptidase IV inhibitor" OR "Gliptin*" OR "DPP4" OR "DPP 4" OR "DPP IV" OR "Alogliptin" OR "Anagliptin" OR "Gemigliptin" OR "Linagliptin" OR "Saxagliptin" OR "Sitagliptin" OR "Teneligliptin" OR "Vildagliptin") and Preprint Citation Index (Exclude – Database) |
| #5 | TS=(“GLP 1" OR "Glucagon Like Peptide 1" OR "glucagon like peptide i" OR "insulinotropin" OR "Efpeglenatide" OR "Dulaglutide" OR "Semaglutide" OR "Taspoglutide" OR "Albiglutide" OR "tirzepatide" OR "Liraglutide" OR "Lixisenatide" OR "Exenatide") and Preprint Citation Index (Exclude – Database) |
| #6 | TS=(“Randomized Controlled Trials as Topic" OR "random*" OR "placebo" OR "control*" OR "randomized controlled trial ") and Preprint Citation Index (Exclude – Database) |
| #7 | #1 AND #2 AND (#3 OR #4 OR #5) AND #6 and Preprint Citation Index (Exclude – Database) |

**Cochrane**

| **No.** | **Query** |
| --- | --- |
| #1 | ('adult onset diabetes' OR 'diabetes mellitus type 2' OR 'diabetes mellitus type ii' OR 'diabetes type 2' OR 'diabetes type II' OR 'insulin independent diabetes' OR 'ketosis resistant diabetes mellitus' OR 'Maturity Onset Diabetes' OR 'MODY' OR 'NIDDM' OR 'non insulin dependent diabetes' OR 'noninsulin dependent diabetes' OR 'Slow Onset Diabetes Mellitus' OR 'Stable Diabetes Mellitus' OR 'T2DM' OR 'TIIDM' OR 'type 2 diabetes' OR 'type II diabetes'):ti,ab,kw |
| #2 | MeSH descriptor: [Diabetes Mellitus, Type 2] explode all trees |
| #3 | ('Chronic Kidney Disease*' OR 'chronic kidney disorder' OR 'chronic kidney failure' OR 'Chronic Kidney Insufficienc*' OR 'chronic nephropathy' OR 'Chronic Renal Disease*' OR 'chronic renal failure' OR 'Chronic Renal Insufficienc*' OR 'kidney chronic failure'):ti,ab,kw |
| #4 | MeSH descriptor: [Renal Insufficiency, Chronic] explode all trees |
| #5 | ('Gliflozin*' OR 'sodium dependent glucose cotransporter 2 inhibitor' OR 'sodium glucose cotransporter 2 inhibitor' OR 'sodium glucose co-transporter 2 inhibitor' OR 'Sodium Glucose Transporter 2 Inhibitor*' OR 'SGLT2' OR 'SGLT 2' OR 'canagliflozin' OR 'dapagliflozin' OR 'Empagliflozin' OR 'ipragliflozin' OR 'tofogliflozin' OR 'luseogliflozin' OR 'remogliflozin' OR 'ertugliflozin' OR 'sotagliflozin' OR 'bexagliflozin'):ti,ab,kw |
| #6 | MeSH descriptor: [Sodium-Glucose Transporter 2 Inhibitors] explode all trees |
| #7 | ('Dipeptidyl Peptidase 4 Inhibitor*' OR 'Dipeptidyl Peptidase IV Inhibitor*' OR 'dipeptidylpeptidase 4 inhibitor' OR 'dipeptidylpeptidase IV inhibitor' OR 'Gliptin*' OR 'DPP4' OR 'DPP 4' OR 'DPP IV' OR 'Alogliptin' OR 'Anagliptin' OR 'Gemigliptin' OR 'Linagliptin' OR 'Saxagliptin' OR 'Sitagliptin' OR 'Teneligliptin' OR 'Vildagliptin'):ti,ab,kw |
| #8 | MeSH descriptor: [Dipeptidyl-Peptidase IV Inhibitors] explode all trees |
| #9 | ('GLP 1' OR 'Glucagon Like Peptide 1' OR 'glucagon like peptide i' OR 'insulinotropin' OR 'Efpeglenatide' OR 'Dulaglutide' OR 'Semaglutide' OR 'Taspoglutide' OR 'Albiglutide' OR 'tirzepatide' OR 'Liraglutide' OR 'Lixisenatide' OR 'Exenatide'):ti,ab,kw |
| #10 | MeSH descriptor: [Glucagon-Like Peptide 1] explode all trees |
| #11 | ('Randomized Controlled Trials as Topic' OR 'random*' OR 'placebo' OR 'control*' OR 'randomized controlled trial '):ti,ab,kw |
| #12 | MeSH descriptor: [Randomized Controlled Trials as Topic] explode all trees |
| #13 | (#1 OR #2) AND (#3 OR #4) AND (#5 OR #6 OR #7 OR #8 OR #9 OR #10) AND (#11 OR #12) |

**Table S2** Baseline and outcome

| **Author** | **Year** | **Country** | **Study number** | **Intervention** | **Sample size** | **Age** | **Male/Female** | **Follow-up** | **CKD definition** | **Outcome** | **Key findings** |
| --- | --- | --- | --- | --- | --- | --- | --- | --- | --- | --- | --- |
| Perkovic et al. | 2024 | Multiple cpuntries | NCT03819153 | Semaglutide 1.0mg/wk | 1767 | 66.6±9.0 | 1248/519 | 3.4 years | eGFR 50-75 & UACR>300 to <5000; or eGFR 25 to <50 & UACR>100 to <5000 | Composite renal outcomes, Cardiovascular death, MACEs, All-cause mortality, Hypoglycemia | Semaglutide significantly reduced the risks of composite renal outcomes (HR 0.76, P=0.0003), cardiovascular death (HR 0.71), MACEs (HR 0.82, P=0.029), and all-cause mortality (HR 0.80, P=0.01). |
|  |  |  |  | Placebo | 1766 | 66.7±9.0 | 1216/550 | 3.4 years |  |  |  |
| Sivalingam et al. | 2024 | Denmark | NCT04061200 | Semaglutide 1.0mg/wk | 30 | 70.5±6.8 | 24/6 | 26 weeks | eGFR≥30 & UACR>100 | All-cause mortality, Hypoglycemia, MACEs | Semaglutide did not significantly reduce UACR (P=0.15) but significantly lowered HbA1c levels (P=0.003). All-cause mortality was 3.3% (1 case) in the semaglutide group. The incidence of hypoglycemia was 3.3% in the semaglutide group and 7% in the placebo group. |
|  |  |  |  | Placebo | 30 | 69.4±9.1 | 23/7 | 26 weeks |  |  |  |
| Pollock et al. | 2019 | Multiple cpuntries | NCT02547935 | Dapagliflozin 10mg | 145 | 64.7±8.6 | 102/43 | 24 weeks | eGFR 25-75 & UACR 30-3500 | AEs, Hypoglycemia, Composite renal outcomes, All-cause mortality | After 24 weeks of treatment, the dapagliflozin group showed a significant reduction in UACR by 21% compared to the placebo group (95% CI −34.1 to −5.2; P=0.011). The dapagliflozin + saxagliptin group demonstrated an even greater reduction in UACR (38%; 95% CI −48.2 to −25.8; p<0.0001) and significantly improved glycemic control (HbA1c reduction by 0.58%; P<0.0001). Adverse event rates were similar across groups (54%–68%), but the incidence of mild hypoglycemia was higher in the combined therapy group (33%) than in the placebo group (20%). Severe hypoglycemia and all-cause mortality were rare and balanced across groups (1 all-cause death each in the dapagliflozin group and the combined therapy group; 0 in the placebo group). |
|  |  |  |  | Dapagliflozin 10mg+Saxagliptin 2.5mg | 155 | 64.0±9.2 | 110/45 | 24 weeks |  |  |  |
|  |  |  |  | Placebo | 148 | 64.7±8.5 | 105/43 | 24 weeks |  |  |  |
| Beek et al. | 2023 | Netherlands | EUCTR2017-004709-42-NL | Dapagliflozin 10mg | 20 | 70.5±5.1 | 16/4 | 6 weeks | eGFR>30 & UACR>30.9 to ≤884 | AEs, Hypoglycemia | Hypoglycemia was rare (1 case each in monotherapy groups; 0 in the combined therapy group); gastrointestinal reactions were more frequent during treatment with exenatide; urinary tract infections occurred in the dapagliflozin group. |
|  |  |  |  | Exenatide 2mg/wk | 20 | 70.5±5.1 | 16/4 | 6 weeks |  |  |  |
|  |  |  |  | Dapagliflozin 10mg+Exenatide 2mg/wk | 20 | 70.5±5.1 | 16/4 | 6 weeks |  |  |  |
| Perkovic et al. | 2019 | Multiple cpuntries | NCT02065791 | Canagliflozin 100mg | 2202 | 62.9±9.2 | 1440/762 | 2.62 years | eGFR 30 to <90 & UACR>300 to 5000 | Cardiovascular death, MACEs, Composite renal outcomes, All-cause mortality, AEs | The canagliflozin group showed a 30% reduction in the risk of the primary outcome (composite renal outcomes) (P=0.00001), a 20% reduction in the risk of MACEs (P=0.01), a 17% reduction in the risk of all-cause mortality (HR 0.83), and a 22% reduction in the risk of cardiovascular death (P=0.05). |
|  |  |  |  | Placebo | 2199 | 63.2±9.2 | 1467/732 | 2.62 years |  |  |  |
| Tuttle et al. | 2018 | Multiple cpuntries | NCT01621178 | Dulaglutide 1.5mg/wk | 192 | 64.7±8.8 | 104/88 | 52 weeks | eGFR 15-59 | AEs, Composite renal outcomes, MACEs, All-cause mortality, Cardiovascular death, Hypoglycemia | Dulaglutide significantly reduced the risk of severe hypoglycemia (0% in the 1.5 mg group vs. 7% in the insulin glargine group, p=0.0003; the incidence of symptomatic hypoglycemia reduced by approximately 50%). All-cause mortality (1%-4%) and cardiovascular death (1%-3%) were rare with no significant differences among the three groups. The incidence of serious adverse events was similar (20%-27%) among groups. The incidence of gastrointestinal adverse reactions (20% for nausea, 17% for diarrhea, and 14% for vomiting) and the rate of discontinuation due to adverse events (10%-13%) were higher in the dulaglutide group than in the glargine insulin group (6%). |
|  |  |  |  | Dulaglutide 0.75mg/wk | 190 | 64.7±8.6 | 104/86 | 52 weeks |  |  |  |
|  |  |  |  | Glargine | 194 | 64.3±8.4 | 93/101 | 52 weeks |  |  |  |
| Wada et al. | 2022 | Japan | NCT03436693 | Canagliflozin 100mg | 154 | 62.5±10.5 | 115/39 | 104 weeks | eGFR≥30 to <90 & UACR≥300 to ≤5000 | MACEs, Composite renal outcomes, Cardiovascular death, All-cause mortality, AEs, Hypoglycemia | The canagliflozin group showed a significant reduction in the incidence of eGFR decline ≥ 30% compared to the placebo group, along with a significant reduction in UACR, with effects persisting through week 104. Incidences of MACEs, cardiovascular death, all-cause mortality, and composite renal outcomes were low, with no statistically significant differences between groups. Overall adverse event rates were similar, and no severe hypoglycemic events were reported. |
|  |  |  |  | Placebo | 154 | 62.4±11.1 | 129/25 | 104 weeks |  |  |  |
| Rosenstock et al. | 2019 | Multiple cpuntries | NCT01897532 | Linagliptin 5mg | 3494 | 66.1±9.1 | 2148/1346 | 2.2 years | eGFR 45-75 & UACR>200; or eGFR 15-45 | MACEs, Composite renal outcomes, All-cause mortality, Cardiovascular death, AEs, Hypoglycemia | Linagliptin showed non-inferiority to placebo for reducing MACEs (HR 1.02, 95% CI 0.89-1.17) but did not significantly improve composite renal outcomes (HR 1.04, 95% CI 0.89-1.22). The incidence rates of all-cause mortality, cardiovascular death, hypoglycemia, and adverse events in the linagliptin group were similar to those in the placebo group, with no additional risk of heart failure. |
|  |  |  |  | Placebo | 3485 | 65.6±9.1 | 2242/1243 | 2.2 years |  |  |  |
| Yale et al. | 2013 | Multiple cpuntries | NCT01064414​ | Canagliflozin 100mg | 90 | 69.5±8.2 | 58/32 | 26 weeks | eGFR≥30 to <50 | AEs, All-cause mortality | Canagliflozin 100 mg and 300 mg significantly improved glycemic control over 26 weeks (HbA1c reduced by 0.33% and 0.44% compared to the placebo group, respectively) and reduced body weight and blood pressure. All-cause mortality rates were 1.1% (100 mg group), 1.1% (placebo group), and 0% (300 mg group). |
|  |  |  |  | Canagliflozin 300mg | 89 | 67.9±8.2 | 48/41 | 26 weeks |  |  |  |
|  |  |  |  | Placebo | 90 | 68.2±8.4 | 57/33 | 26 weeks |  |  |  |
| Yale et al. | 2014 | Multiple cpuntries | NCT01064414​ | Canagliflozin 100mg | 90 | 69.5±8.2 | 58/32 | 52 weeks | eGFR≥30 to <50 | AEs, All-cause mortality | Canagliflozin 100 mg and 300 mg continuously reduced HbA1c (by 0.27% and 0.41% more than the placebo group, respectively), body weight, and blood pressure over 52 weeks. Overall adverse event rates were similar across the three groups (80.9%-86.7%). All-cause mortality rates were 2.2% in the placebo group, 4.4% in the 100 mg group, and 0% in the 300 mg group, all considered unrelated to study drugs. |
|  |  |  |  | Canagliflozin 300mg | 89 | 67.9±8.2 | 48/41 | 52 weeks |  |  |  |
|  |  |  |  | Placebo | 90 | 68.2±8.4 | 57/33 | 52 weeks |  |  |  |
| Parker et al. | 2022 | UK and Germany | NCT03550378 | Cotadutide 50μg(4 days)→100μg(7 days)→200μg(7 days)→300μg(14 days) | 21 | 71.1±7.4 | 12/9 | 32 days | eGFR 30-59 | AEs, All-cause mortality, Hypoglycemia | Short-term (32-day) cotadutide treatment demonstrated acceptable overall safety, though treatment-emergent adverse events were more frequent than with placebo (95.2% vs 65.0%), primarily gastrointestinal reactions (42.9% for nausea, 28.6% for vomiting). The proportion of time spent with clinically significant hypoglycemia (<3.0 mmol/L) was significantly increased (2.01% vs 0.66%, P=0.010), but no severe hypoglycemic events occurred, and symptoms resolved by reducing insulin dosage. |
|  |  |  |  | Placebo | 20 | 70.9±4.7 | 9/11 | 32 days |  |  |  |
| Fioretto et al. | 2018 | Multiple cpuntries | NCT02413398 | Dapagliflozin 10mg | 160 | 65.3±66.0 | 91/69 | 24 weeks | eGFR 45-59 | AEs, All-cause mortality, Hypoglycemia | The incidence of adverse events was slightly lower in the dapagliflozin group (41.9% vs 47.8%), with fewer serious adverse events (5.6% vs 8.7%). No fracture, amputation, or diabetic ketoacidosis events were reported. No deaths occurred in either group. The incidence of hypoglycemia was comparable (12.5% vs 13.7%), with no severe hypoglycemic events and no discontinuations due to hypoglycemia. |
|  |  |  |  | Placebo | 161 | 66.2±68.0 | 91/70 | 24 weeks |  |  |  |
| Hameed et al. | 2024 | India | NA | Empagliflozin 10mg | 51 | 62.69±8.63 | 25/26 | 12 months | eGFR<60 | Hypoglycemia | Hypoglycemia: The incidence of hypoglycemia in the empagliflozin group (43.1%) was higher than that in the linagliptin group (31.5%). Hypoglycemia was one of the most common adverse events, but symptoms were alleviated by reducing insulin dosage. |
|  |  |  |  | Linagliptin 5mg | 54 | 64.41±8.56 | 24/30 | 12 months |  |  |  |
| Ferreira et al. | 2013 | Multiple cpuntries | NCT00509236 | Sitagliptin 25mg | 64 | 60.5±9.1 | 40/24 | 54 weeks | eGFR<15 | AEs, All-cause mortality, Hypoglycemia | Compared with glipizide, sitagliptin significantly reduced the risk of severe hypoglycemia (0% vs 7.7%) and the incidence of symptomatic hypoglycemia (6.3% vs 10.8%). There were no significant differences in the incidence rates of overall adverse events (82.8% vs 80.0%) or mortality (6.3% vs 9.2%) between the two groups. |
|  |  |  |  | Glipizide 20mg | 65 | 58.5±9.9 | 37/28 | 54 weeks |  |  |  |
| Cherney et al. | 2021 | Multiple cpuntries | NCT03242018 | Sotagliflozin 200mg | 92 | 66.8±10.0 | 44/48 | 52 weeks | eGFR≥15 to ＜30 | AEs, All-cause mortality, Hypoglycemia, MACEs, Cardiovascular death, Composite renal outcomes | Compared to the placebo group, the sotagliflozin groups showed reduced incidence rates of MACEs (3.2%/4.4% vs 12.9%), cardiovascular death (0%/1.1% vs 5.4%), and all-cause mortality (3.2%/2.2% vs 5.4%). The overall incidence of hypoglycemia was similar among groups (38.9%-40.9%). Severe hypoglycemia was observed only in the sotagliflozin 200 mg group (3.2%). The incidence of adverse events was similar among groups (81.1%-86.2%). |
|  |  |  |  | Sotagliflozin 400mg | 92 | 67.3±9.6 | 49/43 | 52 weeks |  |  |  |
|  |  |  |  | Placebo | 93 | 68.0±8.3 | 42/51 | 52 weeks |  |  |  |
| Barnett et al. | 2014 | Multiple cpuntries | NCT01164501​ | Empagliflozin 10mg | 98 | 63.2±8.5 | 60/38 | 52 weeks | eGFR≥60 to <90 | AEs, All-cause mortality, Hypoglycemia | AEs: the incidence of AEs was similar between the empagliflozin group and the placebo group among patients with stage 2-3 CKD (80%-87%); the incidence of AEs was slightly higher in the empagliflozin group than in the placebo group among patients with stage 4 CKD (91.9% vs 83.8%). Mortality: No deaths occurred in patients with stage 2 CKD; the incidence of death was 0.5% in both the empagliflozin group and the placebo group among patients with stage 3 CKD; the incidence of death was 0% in the empagliflozin group and 5.4% in the placebo group among patients with stage 4 CKD. Hypoglycemia: the incidence of hypoglycemia was similar between the empagliflozin group and the placebo group across CKD stages; the incidence of severe hypoglycemia requiring assistance was ≤2.7%. |
|  |  |  |  | Empagliflozin 25mg | 97 | 62.0±8.4 | 61/36 | 52 weeks | eGFR≥60 to <90 |  |  |
|  |  |  |  | Placebo | 95 | 62.6±8.1 | 56/39 | 52 weeks | eGFR≥60 to <90 |  |  |
|  |  |  |  | Empagliflozin 25mg | 187 | 64.6±8.9 | 107/80 | 52 weeks | eGFR≥30 to <60 |  |  |
|  |  |  |  | Placebo | 187 | 65.1±8.2 | 106/81 | 52 weeks | eGFR≥30 to <60 |  |  |
|  |  |  |  | Empagliflozin 25mg | 37 | 65.4±10.2 | 21/16 | 52 weeks | eGFR≥15 to <30 |  |  |
|  |  |  |  | Placebo | 37 | 62.9±11.9 | 19/18 | 52 weeks | eGFR≥15 to <30 |  |  |
| Leiter et al. | 2014 | Multiple cpuntries | NCT01098539 | Albiglutide 30mg/50mg/wk | 249 | 63.2±8.37 | 136/113 | 52 weeks | eGFR≥15 to ≤89 | AEs, Hypoglycemia | AEs: The overall incidence of AEs was similar between groups (83.5% vs 83.3%); the incidence of serious AEs was similar (12.9% vs 14.6%); the incidence of gastrointestinal AEs was slightly higher in the albiglutide group (31.7% vs 25.2%, primarily diarrhea). Hypoglycemia: The albiglutide group showed a higher overall incidence of hypoglycemia (24.1% vs 15.9%), but a lower incidence of severe hypoglycemia (0.4% vs 1.6%). |
|  |  |  |  | Sitagliptin 25mg/50mg/100mg | 246 | 63.5±9.02 | 130/116 | 52 weeks |  |  |  |
| Kaku et al. | 2020 | Japan | NCT02512068 | Trelagliptin 25mg/wk | 55 | 65.8±10.28 | 38/17 | 12 weeks | Ccr<30; or ESRD | AEs, All-cause mortality | AEs: The incidence of AEs was 72.7% in the trelagliptin group and 61.5% in the placebo group. All-cause mortality: No deaths occurred during the study period (0%). No clinically significant hypoglycemic events were observed. The drug was well tolerated in patients with severe renal failure (Ccr<30) and patients receiving dialysis. |
|  |  |  |  | Placebo | 52 | 65.8±10.46 | 39/13 | 12 weeks |  |  |  |
| Grunberger et al. | 2018 | Multiple cpuntries | NCT01986855 | Ertugliflozin 5mg | 158 | 66.7±8.3 | 84/74 | 26 weeks | eGFR 30-60 | AEs, All-cause mortality, Hypoglycemia | AEs: The overall incidence of AEs was similar across groups (74%-85%); drug-related AEs were more frequent in the ertugliflozin 5 mg group (24.7%) than in the placebo group (14.9%). All-cause mortality: The incidence of all-cause mortality was comparable across groups (1.9% vs 1.9% vs 2.6%). Hypoglycemia: The incidence of symptomatic hypoglycemia was similar across groups (18%-25%); in the cohort of patients with stage 3A CKD, the ertugliflozin 15 mg group showed a lower risk of hypoglycemia than the placebo group (12.4% vs 21.2%). |
|  |  |  |  | Ertugliflozin 15mg | 155 | 67.5±8.5 | 75/80 | 26 weeks |  |  |  |
|  |  |  |  | Placebo | 154 | 67.5±8.9 | 72/82 | 26 weeks |  |  |  |
|  |  |  |  | Ertugliflozin 5mg | 158 | 66.7±8.3 | 84/74 | 52 weeks |  |  |  |
|  |  |  |  | Ertugliflozin 15mg | 155 | 67.5±8.5 | 75/80 | 52 weeks |  |  |  |
|  |  |  |  | Placebo | 154 | 67.5±8.9 | 72/82 | 52 weeks |  |  |  |
| Kohan et al. | 2014 | Multiple cpuntries | NCT00663260​ | Dapagliflozin 5mg | 83 | 66±8.9 | 55/28 | 104 weeks | eGFR 30-59 | AEs, All-cause mortality, Hypoglycemia | AEs: The overall incidence of AEs was similar across groups (91%-96%). All-cause mortality: The incidences of all-cause mortality were 6.0%, 2.4%, and 3.5%, respectively; no drug-related deaths occurred. Hypoglycemia: Severe hypoglycemia was rare (0%-4.8%); dapagliflozin did not increase the overall risk of hypoglycemia (38%-46% vs 51.2%). |
|  |  |  |  | Dapagliflozin 10mg | 85 | 68±7.7 | 56/29 | 104 weeks |  |  |  |
|  |  |  |  | Placebo | 84 | 67±8.6 | 53/31 | 104 weeks |  |  |  |
| Nowicki et al. | 2011 | Multiple cpuntries | NCT00614939 | Saxagliptin 2.5mg | 85 | 67 | 50/35 | 52 weeks | CrCl<50 | AEs, All-cause mortality, Hypoglycemia | AEs: The overall incidence of AEs was similar between groups (75% vs 71%). Hypoglycemia: No increased risk was observed in the saxagliptin group compared to the placebo group (28% vs 29%). All-cause mortality: 3.5% vs 4.7% (all unrelated to treatment). |
|  |  |  |  | Placebo | 85 | 67 | 51/34 | 52 weeks |  |  |  |
| Chacra et al. | 2017 | Multiple cpuntries | NCT01698775 | Omarigliptin 25mg/12.5mg/wk | 107 | 65.9±9.4 | 68/39 | 24 weeks | eGFR<60 | AEs, All-cause mortality, Hypoglycemia | AEs: The overall incidence of AEs was similar between groups (66% vs 70%). Hypoglycemia: The incidence of symptomatic hypoglycemia was similar (17% vs 15%, P=0.71). All-cause mortality: 1 all-cause death in each group (0.9%), both unrelated to treatment. |
|  |  |  |  | Placebo | 106 | 64.5±9.7 | 63/43 | 24 weeks |  |  |  |
| Rajesh et al. | 2023 | India | NA | ADD+Enalapril 2.5mg+Sitagliptin 100mg | 30 | 63.46±5.61 | 21/9 | 24 weeks | eGFR>60 & UACR>30 | Hypoglycemia | The sitagliptin group showed a significantly lower incidence of AEs compared to the control group (3.3% vs 13.3%). All events were mild, with no severe hypoglycemia. |
|  |  |  |  | ADD+Enalapril 2.5mg | 30 | 63.21±5.99 | 24/6 | 24 weeks |  |  |  |
| Selvarajah et al. | 2024 | Multiple cpuntries | NCT04515849 | Cotadutide 100μg | 52 | 67.2±7.3 | 43/9 | 26 weeks | eGFR≥20 to <90 & UACR>50 | AEs, All-cause mortality, Hypoglycemia | AEs: Cotadutide 600 μg demonstrated comparable safety to semaglutide (incidence of AEs: 10.4% vs 8.9%); AEs were primarily gastrointestinal reactions (nausea 27% vs 24%). All-cause mortality: 2 all-cause deaths (3.6%) in the cotadutide 100 μg group (acute myocardial infarction and suspected suicide), 0 in other groups; all determined to be unrelated to treatment. |
|  |  |  |  | Cotadutide 300μg | 49 | 65.7±8.8 | 46/3 | 26 weeks |  |  |  |
|  |  |  |  | Cotadutide 600μg | 51 | 66.1±7.4 | 41/10 | 26 weeks |  |  |  |
|  |  |  |  | Placebo | 51 | 69.5±7.3 | 38/13 | 26 weeks |  |  |  |
|  |  |  |  | Semaglutide 1mg/wk | 45 | 67.0±7.8 | 33/12 | 26 weeks |  |  |  |
| Allegretti et al. | 2019 | Multiple cpuntries | NCT02836873 | Bexagliflozin 20mg | 157 | 69.3±8.36 | 92/65 | 24 weeks | eGFR 30-59 | AEs, All-cause mortality, Hypoglycemia, MACEs | Bexagliflozin demonstrated favorable safety comparable to placebo, with similar overall incidence rates of treatment-emergent AEs (69.4% vs. 67.7%) and serious AEs (7.0% vs. 5.8%). Notably, no deaths occurred in either group during the 24-week follow-up period, and the incidence rate of hypoglycemia was comparable between groups (24.8% vs. 24.5%), and no severe hypoglycemic episodes occurred. |
|  |  |  |  | Placebo | 155 | 69.9±8.29 | 104/51 | 24 weeks |  |  |  |
| Lukashevich et al. | 2011 | Multiple cpuntries | NA | Vildagliptin 50mg | 165 | 67.7±8.8 | 96/69 | 24 weeks | eGFR≥30 to <50 | AEs, All-cause mortality, Hypoglycemia | The overall incidence of AEs was similar between the vildagliptin and placebo groups (among patients with moderate renal impairment: 67.5% vs. 72.9%; among patients with severe renal impairment: 72.6% vs. 74.2%). All-cause mortality rates were comparable between groups (1.8% in vildagliptin group vs. 2.2% in placebo group), with no deaths considered related to study drugs. For hypoglycemia, the overall incidence was slightly higher in the vildagliptin group than in the placebo group (among patients with moderate renal impairment: 17.2% vs. 11.6%; among patients with severe renal impairment: 15.3% vs. 12.4%). |
|  |  |  |  | Placebo | 129 | 69.7±7.3 | 80/49 | 24 weeks | eGFR≥30 to <50 |  |  |
|  |  |  |  | Vildagliptin 50mg | 124 | 64.1±9.2 | 65/59 | 24 weeks | eGFR<30 |  |  |
|  |  |  |  | Placebo | 97 | 64.5±10.8 | 53/44 | 24 weeks | eGFR<30 |  |  |
| Bhatt et al. | 2021 | Multiple cpuntries | NCT03315143 | Sotagliflozin 200mg/400mg | 5292 | 68.7±8.2​ | 2945/2347 | 16 months | eGFR 25-60 | Cardiovascular death, Composite renal outcomes, All-cause mortality, MACEs | Sotagliflozin significantly reduced the risk of MACEs (HR 0.84, 95% CI 0.72–0.99) but did not significantly reduce the risks of cardiovascular death (HR 0.90, 95% CI 0.73–1.12), composite renal outcomes (HR 0.71, 95% CI 0.46–1.08), or all-cause mortality (HR 0.99, 95% CI 0.83–1.18). Regarding safety, the incidence rates of diarrhea, genital mycotic infections, volume depletion, and diabetic ketoacidosis were higher in the sotagliflozin group than in the placebo group, but the incidence rate of severe hypoglycemia was similar between groups. |
|  |  |  |  | Placebo | 5292 | 68.7±8.2​ | 2885/2407 | 16 months |  |  |  |
| Davies et al. | 2014 | Multiple cpuntries | NCT01620489 | Liraglutide 1.8mg | 140 | 68.0±8.3 | 75/65 | 26 weeks | eGFR 30-59 | AEs, All-cause mortality | The liraglutide group showed a higher incidence of AEs than the placebo group (76.4% vs 68.6%). Liraglutide did not increase the risk of hypoglycemia (20.7% vs 26.3%). There were 4 all-cause deaths in the liraglutide group and 1 in the placebo group, both determined to be unrelated to the drug. |
|  |  |  |  | Placebo | 137 | 66.3±8.0 | 65/72 | 26 weeks |  |  |  |
| Haneda et al. | 2016 | Japan | JapicCTI-111543 | Luseogliflozin 2.5mg | 95 | 67.9±8.9 | 72/23 | 24 weeks | eGFR≥30 to <60 | AEs, All-cause mortality, Hypoglycemia | Luseogliflozin significantly reduced HbA1c (-0.19%, P=0.041), fasting blood glucose (-7.8 mg/dL), and body weight (-1.28 kg). Safety: The luseogliflozin group showed an overall incidence of AEs similar to that of the placebo group (67% vs 62%) and a lower risk of hypoglycemia (3.2% vs 6.0%). No serious events were reported. One death occurred (unrelated to the drug). |
|  |  |  |  | Placebo | 50 | 68.4±8.9 | 39/11 | 24 weeks |  |  |  |
| Wanner et al. | 2016 | Multiple cpuntries | NCT01131676​​ | Empagliflozin 10mg/25mg | 1212 | 67.1±7.6 | 816/396 | 3.1 years | eGFR 30-59 | Composite renal outcomes, AEs, All-cause mortality, Hypoglycemia | Compared with placebo, empagliflozin significantly reduced the risk of composite renal outcomes by 39% (HR 0.61, 95% CI 0.53-0.70, P<0.001) and lowered the risk of all-cause mortality (HR 0.68, 95% CI 0.57-0.82). The overall incidences of adverse events and hypoglycemia in the empagliflozin group were similar to those in the placebo group. |
|  |  |  |  | Placebo | 607 | 67.1±8.2 | 418/189 | 3.1 years | eGFR 30-59 |  |  |
|  |  |  |  | Empagliflozin 10mg/25mg | 3473 | 61.7±8.5 | 2518/955 | 3.1 years | eGFR≥60 |  |  |
|  |  |  |  | Placebo | 1726 | 61.9±8.6 | 1262/464 | 3.1 years | eGFR≥60 |  |  |

**Table S3** Comparison of MACEs in patients with type 2 diabetes and chronic kidney disease

| Bexagliflozin | 0.22 (0.01, 2.01) | 0.27 (0.01, 2.47) | 0.27 (0.01, 2.42) | 0.23 (0.01, 2.05) | 0.2 (0.01, 1.83) |
| --- | --- | --- | --- | --- | --- |
| 4.51 (0.5, 127.96) | Canagliflozin | 1.22 (0.96, 1.56) | 1.21 (1.02, 1.43) | 1.02 (0.8, 1.29) | 0.92 (0.74, 1.14) |
| 3.7 (0.41, 105.07) | 0.82 (0.64, 1.04) | Linagliptin | 0.99 (0.83, 1.18) | 0.83 (0.65, 1.06) | **0.75 (0.6, 0.94)** |
| 3.73 (0.41, 105.45) | **0.83 (0.7, 0.98)** | 1.01 (0.85, 1.21) | Placebo | **0.84 (0.71, 1)** | **0.76 (0.66, 0.87)** |
| 4.43 (0.49, 125.66) | 0.98 (0.77, 1.25) | 1.2 (0.94, 1.54) | 1.19 (1, 1.41) | Semaglutide | 0.9 (0.73, 1.12) |
| 4.92 (0.55, 139.98) | 1.09 (0.88, 1.35) | 1.33 (1.07, 1.67) | 1.32 (1.15, 1.51) | 1.11 (0.89, 1.38) | Sotagliflozin |

Note: The bolded data represent significant differences; MACE=major adverse cardiovascular event

**Table S4** Comparison of composite renal outcomes in patients with type 2 diabetes and chronic kidney disease

| Canagliflozin | 0.99 (0.24, 3.54) | 2.46 (0.91, 7.38) | 0.85 (0.59, 1.23) | 1.59 (1.25, 2.03) | 1.49 (1.23, 1.81) | 1.2 (0.95, 1.52) | 1.23 (0.83, 1.84) |
| --- | --- | --- | --- | --- | --- | --- | --- |
| 1.01 (0.28, 4.1) | Dapagliflozin | 2.49 (0.84, 9.28) | 0.86 (0.24, 3.56) | 1.61 (0.45, 6.45) | 1.51 (0.43, 6.02) | 1.22 (0.34, 4.87) | 1.25 (0.34, 5.21) |
| 0.41 (0.14, 1.1) | 0.4 (0.11, 1.19) | Dapagliflozin_Saxagliptin | **0.35 (0.11, 0.97)** | 0.65 (0.22, 1.74) | 0.61 (0.21, 1.61) | 0.49 (0.16, 1.31) | 0.5 (0.16, 1.42) |
| 1.17 (0.81, 1.7) | 1.16 (0.28, 4.24) | 2.89 (1.03, 8.94) | Empagliflozin | 1.87 (1.32, 2.64) | 1.75 (1.28, 2.4) | 1.41 (1, 1.98) | 1.45 (0.91, 2.32) |
| **0.63 (0.49, 0.8)** | 0.62 (0.15, 2.22) | 1.55 (0.57, 4.61) | **0.54 (0.38, 0.76)** | Linagliptin | 0.94 (0.81, 1.09) | **0.76 (0.62, 0.92)** | 0.78 (0.53, 1.13) |
| **0.67 (0.55, 0.81)** | 0.66 (0.17, 2.33) | 1.65 (0.62, 4.86) | **0.57 (0.42, 0.78)** | 1.07 (0.92, 1.24) | Placebo | **0.81 (0.71, 0.92)** | 0.83 (0.58, 1.18) |
| 0.83 (0.66, 1.05) | 0.82 (0.21, 2.91) | 2.04 (0.76, 6.08) | **0.71 (0.5, 1)** | 1.32 (1.08, 1.61) | 1.24 (1.09, 1.41) | Semaglutide | 1.03 (0.71, 1.49) |
| 0.81 (0.54, 1.21) | 0.8 (0.19, 2.97) | 2 (0.7, 6.2) | 0.69 (0.43, 1.1) | 1.29 (0.88, 1.88) | 1.21 (0.85, 1.71) | 0.97 (0.67, 1.41) | Sotagliflozin |

Note: The bolded data represent significant differences.

**Table S5** Comparison of all-cause mortality in patients with Type 2 diabetes and chronic kidney disease

| Bexagliflozin | 0.83 (0.02, 28.48) | 1.25 (0.03, 57.72) | 0.71 (0.02, 28.09) | 1.01 (0.02, 57.32) | 0.71 (0.02, 24.59) | 1.22 (0.03, 55.89) | 0.95 (0.03, 32.78) | 5.47 (0.08, 619.55) | 1.32 (0.02, 167.58) | 0.99 (0.01, 142.91) | 0.97 (0.03, 33.37) | 0.7 (0.01, 32.12) | 0.79 (0.02, 27.39) | 0.96 (0.03, 32.83) | 0.9 (0.01, 127.02) | 0.59 (0.01, 25.98) |
| --- | --- | --- | --- | --- | --- | --- | --- | --- | --- | --- | --- | --- | --- | --- | --- | --- |
| 1.21 (0.04, 45.27) | Canagliflozin | 1.48 (0.35, 7.59) | 0.86 (0.3, 2.53) | 1.25 (0.14, 8.9) | 0.86 (0.64, 1.16) | 1.44 (0.38, 7.14) | 1.15 (0.91, 1.46) | 6.06 (0.76, 175.61) | 1.53 (0.12, 48.86) | 1.21 (0.03, 42.99) | 1.17 (0.97, 1.43) | 0.86 (0.16, 4.02) | 0.96 (0.75, 1.24) | 1.16 (0.9, 1.5) | 1.1 (0.03, 37.06) | 0.73 (0.17, 2.86) |
| 0.8 (0.02, 38.4) | 0.68 (0.13, 2.86) | Cotadutide | 0.58 (0.09, 3.44) | 0.82 (0.06, 9.45) | 0.58 (0.11, 2.48) | 0.98 (0.12, 8.17) | 0.78 (0.15, 3.29) | 4.18 (0.3, 154.87) | 1.04 (0.05, 42.58) | 0.8 (0.02, 37.13) | 0.8 (0.16, 3.33) | 0.57 (0.06, 4.76) | 0.65 (0.13, 2.74) | 0.79 (0.15, 3.32) | 0.73 (0.01, 32.36) | 0.48 (0.06, 3.48) |
| 1.41 (0.04, 59.27) | 1.16 (0.4, 3.31) | 1.73 (0.29, 11.67) | Dapagliflozin | 1.45 (0.16, 10.53) | 1 (0.34, 2.86) | 1.69 (0.31, 11.14) | 1.34 (0.46, 3.79) | 7.21 (0.68, 230.38) | 1.8 (0.12, 64.82) | 1.41 (0.03, 56.93) | 1.37 (0.47, 3.83) | 0.99 (0.14, 6.38) | 1.12 (0.38, 3.17) | 1.35 (0.46, 3.83) | 1.28 (0.03, 49.64) | 0.84 (0.14, 4.59) |
| 0.99 (0.02, 63.08) | 0.8 (0.11, 7.15) | 1.21 (0.11, 17.49) | 0.69 (0.09, 6.24) | Dapagliflozin_Saxagliptin | 0.68 (0.1, 6.15) | 1.18 (0.11, 16.94) | 0.92 (0.13, 8.19) | 5.22 (0.28, 254.28) | 1.28 (0.05, 68.79) | 0.99 (0.02, 61.26) | 0.94 (0.13, 8.33) | 0.69 (0.05, 9.69) | 0.77 (0.11, 6.86) | 0.93 (0.13, 8.32) | 0.9 (0.01, 52.13) | 0.58 (0.05, 7.38) |
| 1.41 (0.04, 52.67) | 1.17 (0.87, 1.57) | 1.72 (0.4, 8.87) | 1 (0.35, 2.97) | 1.46 (0.16, 10.35) | Empagliflozin | 1.68 (0.45, 8.3) | 1.35 (1.03, 1.75) | 7.07 (0.89, 205.93) | 1.78 (0.14, 56.95) | 1.42 (0.04, 50.51) | 1.37 (1.09, 1.72) | 1 (0.19, 4.72) | 1.12 (0.85, 1.48) | 1.35 (1.02, 1.8) | 1.29 (0.03, 43.1) | 0.85 (0.2, 3.35) |
| 0.82 (0.02, 39.05) | 0.69 (0.14, 2.6) | 1.02 (0.12, 8.22) | 0.59 (0.09, 3.21) | 0.85 (0.06, 9.01) | 0.59 (0.12, 2.23) | Ertugliflozin | 0.8 (0.16, 2.97) | 4.26 (0.31, 149.36) | 1.05 (0.05, 41.49) | 0.82 (0.02, 36.74) | 0.82 (0.17, 3.01) | 0.58 (0.06, 4.48) | 0.67 (0.14, 2.49) | 0.81 (0.16, 3) | 0.74 (0.02, 31.56) | 0.49 (0.06, 3.3) |
| 1.05 (0.03, 38.98) | 0.87 (0.68, 1.1) | 1.28 (0.3, 6.53) | 0.75 (0.26, 2.17) | 1.09 (0.12, 7.68) | **0.74 (0.57, 0.97)** | 1.25 (0.34, 6.14) | Linagliptin | 5.26 (0.67, 152.09) | 1.32 (0.11, 42.13) | 1.05 (0.03, 37.35) | 1.02 (0.89, 1.17) | 0.74 (0.14, 3.47) | 0.83 (0.68, 1.03) | 1.01 (0.81, 1.25) | 0.96 (0.03, 31.93) | 0.63 (0.15, 2.47) |
| 0.18 (0, 12.54) | 0.17 (0.01, 1.31) | 0.24 (0.01, 3.35) | 0.14 (0, 1.46) | 0.19 (0, 3.59) | 0.14 (0, 1.12) | 0.23 (0.01, 3.24) | 0.19 (0.01, 1.5) | Liraglutide | 0.24 (0, 13.19) | 0.18 (0, 12.32) | 0.19 (0.01, 1.53) | 0.13 (0, 1.89) | 0.16 (0.01, 1.25) | 0.19 (0.01, 1.51) | 0.16 (0, 10.51) | 0.11 (0, 1.42) |
| 0.76 (0.01, 62.51) | 0.66 (0.02, 8.09) | 0.97 (0.02, 19.23) | 0.56 (0.02, 8.59) | 0.78 (0.01, 19.72) | 0.56 (0.02, 6.92) | 0.95 (0.02, 18.38) | 0.76 (0.02, 9.29) | 4.15 (0.08, 245.96) | Luseogliflozin | 0.75 (0.01, 59.76) | 0.77 (0.02, 9.41) | 0.54 (0.01, 10.66) | 0.63 (0.02, 7.78) | 0.76 (0.02, 9.35) | 0.68 (0.01, 51.63) | 0.46 (0.01, 8.25) |
| 1.01 (0.01, 149.52) | 0.82 (0.02, 29.8) | 1.25 (0.03, 59.14) | 0.71 (0.02, 29.01) | 1.01 (0.02, 57.14) | 0.7 (0.02, 25.52) | 1.22 (0.03, 58.24) | 0.95 (0.03, 34.01) | 5.42 (0.08, 637.27) | 1.34 (0.02, 163.57) | Omarigliptin | 0.97 (0.03, 34.72) | 0.7 (0.01, 33.5) | 0.79 (0.02, 28.58) | 0.95 (0.03, 34.45) | 0.91 (0.01, 127.41) | 0.59 (0.01, 26.11) |
| 1.03 (0.03, 38.27) | 0.85 (0.7, 1.03) | 1.25 (0.3, 6.39) | 0.73 (0.26, 2.11) | 1.07 (0.12, 7.46) | **0.73 (0.58, 0.92)** | 1.23 (0.33, 6.01) | 0.98 (0.86, 1.13) | 5.15 (0.66, 148.63) | 1.3 (0.11, 41.28) | 1.03 (0.03, 36.59) | Placebo | 0.73 (0.14, 3.39) | **0.82 (0.7, 0.96)** | 0.99 (0.83, 1.17) | 0.94 (0.03, 31.23) | 0.62 (0.15, 2.4) |
| 1.43 (0.03, 74.02) | 1.17 (0.25, 6.26) | 1.76 (0.21, 17.27) | 1.01 (0.16, 7.16) | 1.46 (0.1, 18.59) | 1 (0.21, 5.38) | 1.72 (0.22, 16.42) | 1.35 (0.29, 7.19) | 7.5 (0.53, 286.13) | 1.84 (0.09, 80.3) | 1.43 (0.03, 70.5) | 1.37 (0.29, 7.27) | Saxagliptin | 1.12 (0.24, 6) | 1.35 (0.29, 7.24) | 1.31 (0.03, 59.81) | 0.85 (0.1, 7.1) |
| 1.26 (0.04, 46.69) | 1.04 (0.81, 1.34) | 1.53 (0.37, 7.85) | 0.89 (0.32, 2.62) | 1.3 (0.15, 9.19) | 0.89 (0.68, 1.18) | 1.5 (0.4, 7.4) | 1.2 (0.97, 1.48) | 6.3 (0.8, 181.61) | 1.59 (0.13, 50.39) | 1.26 (0.03, 44.45) | 1.22 (1.04, 1.44) | 0.89 (0.17, 4.18) | Semaglutide | 1.21 (0.95, 1.53) | 1.15 (0.03, 38.44) | 0.75 (0.18, 2.96) |
| 1.04 (0.03, 38.72) | 0.86 (0.66, 1.12) | 1.27 (0.3, 6.54) | 0.74 (0.26, 2.17) | 1.08 (0.12, 7.61) | **0.74 (0.56, 0.98)** | 1.24 (0.33, 6.15) | 0.99 (0.8, 1.24) | 5.22 (0.66, 150.82) | 1.31 (0.11, 41.98) | 1.05 (0.03, 37.01) | 1.01 (0.85, 1.2) | 0.74 (0.14, 3.45) | 0.83 (0.66, 1.05) | Sotagliflozin | 0.95 (0.03, 31.91) | 0.63 (0.15, 2.46) |
| 1.11 (0.01, 169.69) | 0.91 (0.03, 33.68) | 1.37 (0.03, 68.62) | 0.78 (0.02, 32.86) | 1.11 (0.02, 67.01) | 0.78 (0.02, 28.88) | 1.34 (0.03, 65.77) | 1.05 (0.03, 38.74) | 6.07 (0.1, 713.91) | 1.48 (0.02, 188.35) | 1.1 (0.01, 166.29) | 1.07 (0.03, 39.32) | 0.77 (0.02, 38.28) | 0.87 (0.03, 32.43) | 1.05 (0.03, 38.78) | Trelagliptin | 0.65 (0.02, 30.26) |
| 1.68 (0.04, 81.5) | 1.38 (0.35, 5.87) | 2.07 (0.29, 17.47) | 1.2 (0.22, 7.02) | 1.72 (0.14, 19.05) | 1.18 (0.3, 5.05) | 2.03 (0.3, 16.85) | 1.59 (0.4, 6.76) | 8.72 (0.7, 314.69) | 2.17 (0.12, 86.71) | 1.7 (0.04, 76.55) | 1.62 (0.42, 6.83) | 1.18 (0.14, 9.63) | 1.32 (0.34, 5.63) | 1.6 (0.41, 6.78) | 1.53 (0.03, 66.19) | Vildagliptin |

Note: The bold data represent significant differences.

**Table S6** Comparison of AEs in patients with type 2 diabetes and chronic kidney disease

| Bexagliflozin | 0.94  (0.81, 1.1) | 1.13  (0.91, 1.43) | 0.98  (0.83, 1.16) | 1.8 (0.75, 5.09) | 1.22 (0.98, 1.52) | 0.96  (0.82, 1.12) | 0.96  (0.8, 1.15) | 1.47  (0.56, 4.32) | 0.96  (0.83, 1.12) | 1.09  (0.88, 1.34) | 1.07  (0.8, 1.48) | 0.92  (0.72, 1.18) | 0.98  (0.84, 1.14) | 1.04  (0.82, 1.33) | 1.18  (0.91, 1.52) | 0.99  (0.82, 1.2) | 1.16  (0.85, 1.6) | 0.93  (0.77, 1.12) |
| --- | --- | --- | --- | --- | --- | --- | --- | --- | --- | --- | --- | --- | --- | --- | --- | --- | --- | --- |
| 1.06 (0.91, 1.24) | Canagliflozin | 1.2 (1.03, 1.44) | 1.04 (0.97, 1.12) | 1.91 (0.81, 5.36) | 1.3 (1.11, 1.52) | 1.02 (0.99, 1.05) | 1.02 (0.93, 1.13) | 1.56 (0.6, 4.55) | 1.02 (0.99, 1.06) | 1.15 (0.99, 1.34) | 1.13 (0.88, 1.51) | 0.98 (0.81, 1.18) | 1.04 (1.01, 1.06) | 1.1 (0.92, 1.34) | 1.25 (1.02, 1.54) | 1.05 (0.94, 1.19) | 1.22 (0.93, 1.64) | 0.98 (0.88, 1.1) |
| 0.88 (0.7, 1.1) | **0.83 (0.7, 0.97)** | Cotadutide | 0.87 (0.72, 1.03) | 1.59 (0.66, 4.49) | 1.08 (0.85, 1.35) | **0.85 (0.71, 0.99)** | 0.85 (0.69, 1.02) | 1.29 (0.49, 3.84) | **0.85 (0.71, 1)** | 0.96 (0.76, 1.19) | 0.94 (0.7, 1.31) | 0.81 (0.63, 1.04) | 0.86 (0.72, 1.01) | 0.92 (0.71, 1.18) | 1.05 (0.89, 1.18) | 0.87 (0.71, 1.07) | 1.02 (0.74, 1.42) | **0.82 (0.66, 0.99)** |
| 1.02 (0.86, 1.2) | 0.96 (0.89, 1.03) | 1.15 (0.97, 1.39) | Dapagliflozin | 1.83 (0.78, 5.12) | 1.24 (1.06, 1.45) | 0.98 (0.91, 1.04) | 0.98 (0.87, 1.1) | 1.49 (0.57, 4.35) | 0.98 (0.91, 1.05) | 1.1 (0.94, 1.3) | 1.09 (0.84, 1.46) | 0.94 (0.77, 1.14) | 0.99 (0.92, 1.06) | 1.06 (0.87, 1.29) | 1.2 (0.97, 1.49) | 1.01 (0.89, 1.16) | 1.17 (0.89, 1.58) | 0.94 (0.83, 1.07) |
| 0.56 (0.2, 1.33) | 0.52 (0.19, 1.23) | 0.63 (0.22, 1.51) | 0.55 (0.2, 1.28) | Dapagliflozin_Exenatide | 0.68 (0.24, 1.62) | 0.53 (0.19, 1.26) | 0.53 (0.19, 1.26) | 0.82 (0.36, 1.75) | 0.54 (0.19, 1.26) | 0.6 (0.21, 1.44) | 0.6 (0.21, 1.47) | 0.51 (0.18, 1.23) | 0.54 (0.19, 1.27) | 0.58 (0.2, 1.39) | 0.66 (0.23, 1.58) | 0.55 (0.2, 1.31) | 0.64 (0.22, 1.59) | 0.51 (0.18, 1.22) |
| 0.82 (0.66, 1.02) | **0.77 (0.66, 0.9)** | 0.93 (0.74, 1.17) | **0.8 (0.69, 0.94)** | 1.47 (0.62, 4.18) | Dapagliflozin_Saxagliptin | **0.79 (0.67, 0.92)** | **0.78 (0.66, 0.94)** | 1.2 (0.46, 3.54) | **0.79 (0.67, 0.92)** | 0.89 (0.72, 1.1) | 0.87 (0.65, 1.21) | **0.75 (0.59, 0.96)** | **0.8 (0.68, 0.93)** | 0.85 (0.67, 1.09) | 0.96 (0.75, 1.24) | **0.81 (0.67, 0.99)** | 0.94 (0.69, 1.31) | **0.76 (0.63, 0.92)** |
| 1.04 (0.89, 1.21) | 0.98 (0.95, 1.01) | 1.18 (1.01, 1.41) | 1.02 (0.96, 1.1) | 1.87 (0.8, 5.26) | 1.27 (1.09, 1.49) | Empagliflozin | 1 (0.91, 1.1) | 1.53 (0.59, 4.47) | 1 (0.97, 1.03) | 1.13 (0.98, 1.32) | 1.11 (0.87, 1.48) | 0.96 (0.79, 1.16) | 1.01 (1, 1.03) | 1.08 (0.9, 1.31) | 1.23 (1, 1.51) | 1.03 (0.92, 1.17) | 1.2 (0.92, 1.61) | 0.96 (0.86, 1.08) |
| 1.04 (0.87, 1.25) | 0.98 (0.89, 1.08) | 1.18 (0.98, 1.44) | 1.03 (0.91, 1.15) | 1.87 (0.79, 5.28) | 1.28 (1.06, 1.53) | 1 (0.91, 1.1) | Ertugliflozin | 1.53 (0.58, 4.49) | 1.01 (0.91, 1.11) | 1.13 (0.95, 1.35) | 1.11 (0.85, 1.5) | 0.96 (0.78, 1.19) | 1.02 (0.92, 1.12) | 1.08 (0.88, 1.34) | 1.23 (0.98, 1.54) | 1.03 (0.89, 1.21) | 1.2 (0.9, 1.63) | 0.97 (0.83, 1.12) |
| 0.68 (0.23, 1.8) | 0.64 (0.22, 1.67) | 0.77 (0.26, 2.04) | 0.67 (0.23, 1.74) | 1.22 (0.57, 2.8) | 0.83 (0.28, 2.19) | 0.65 (0.22, 1.71) | 0.65 (0.22, 1.71) | Exenatide | 0.66 (0.22, 1.71) | 0.74 (0.25, 1.95) | 0.73 (0.24, 1.97) | 0.63 (0.21, 1.66) | 0.66 (0.23, 1.73) | 0.71 (0.24, 1.88) | 0.8 (0.27, 2.14) | 0.68 (0.23, 1.77) | 0.79 (0.26, 2.13) | 0.63 (0.22, 1.65) |
| 1.04 (0.89, 1.21) | 0.98 (0.94, 1.01) | 1.17 (1, 1.41) | 1.02 (0.95, 1.1) | 1.87 (0.79, 5.25) | 1.27 (1.08, 1.48) | 1 (0.97, 1.03) | 0.99 (0.9, 1.1) | 1.52 (0.58, 4.46) | Linagliptin | 1.13 (0.97, 1.31) | 1.11 (0.86, 1.47) | 0.96 (0.79, 1.16) | 1.01 (0.99, 1.04) | 1.08 (0.89, 1.31) | 1.22 (1, 1.5) | 1.03 (0.92, 1.17) | 1.2 (0.91, 1.61) | 0.96 (0.86, 1.08) |
| 0.92 (0.74, 1.14) | 0.87 (0.74, 1.01) | 1.04 (0.84, 1.31) | 0.91 (0.77, 1.06) | 1.66 (0.69, 4.69) | 1.13 (0.91, 1.39) | 0.88 (0.76, 1.02) | 0.88 (0.74, 1.05) | 1.35 (0.51, 3.98) | 0.89 (0.76, 1.03) | Liraglutide | 0.99 (0.74, 1.35) | 0.85 (0.67, 1.08) | 0.9 (0.77, 1.04) | 0.96 (0.75, 1.22) | 1.09 (0.84, 1.4) | 0.91 (0.76, 1.1) | 1.06 (0.78, 1.47) | 0.85 (0.71, 1.02) |
| 0.93 (0.68, 1.25) | 0.88 (0.66, 1.13) | 1.06 (0.77, 1.44) | 0.92 (0.69, 1.19) | 1.68 (0.68, 4.84) | 1.14 (0.83, 1.53) | 0.9 (0.68, 1.15) | 0.9 (0.67, 1.17) | 1.37 (0.51, 4.11) | 0.9 (0.68, 1.16) | 1.02 (0.74, 1.36) | Luseogliflozin | 0.86 (0.61, 1.18) | 0.91 (0.69, 1.17) | 0.97 (0.69, 1.33) | 1.1 (0.78, 1.52) | 0.93 (0.68, 1.22) | 1.08 (0.73, 1.58) | 0.87 (0.64, 1.14) |
| 1.08 (0.85, 1.38) | 1.02 (0.85, 1.24) | 1.23 (0.96, 1.59) | 1.07 (0.87, 1.3) | 1.95 (0.81, 5.58) | 1.33 (1.04, 1.69) | 1.04 (0.86, 1.26) | 1.04 (0.84, 1.29) | 1.59 (0.6, 4.73) | 1.04 (0.86, 1.26) | 1.18 (0.93, 1.5) | 1.16 (0.85, 1.63) | Omarigliptin | 1.06 (0.88, 1.28) | 1.13 (0.87, 1.48) | 1.28 (0.97, 1.69) | 1.07 (0.86, 1.35) | 1.25 (0.9, 1.77) | 1 (0.81, 1.25) |
| 1.03 (0.88, 1.19) | **0.97 (0.94, 0.99)** | 1.16 (0.99, 1.39) | 1.01 (0.94, 1.08) | 1.84 (0.78, 5.18) | 1.26 (1.07, 1.46) | **0.99 (0.97, 1)** | 0.98 (0.9, 1.09) | 1.51 (0.58, 4.4) | 0.99 (0.96, 1.01) | 1.11 (0.96, 1.3) | 1.1 (0.86, 1.46) | 0.95 (0.78, 1.14) | Placebo | 1.07 (0.89, 1.29) | 1.21 (0.98, 1.48) | 1.01 (0.91, 1.15) | 1.18 (0.9, 1.59) | 0.95 (0.85, 1.06) |
| 0.96 (0.75, 1.22) | 0.91 (0.75, 1.09) | 1.09 (0.85, 1.41) | 0.95 (0.77, 1.15) | 1.73 (0.72, 4.92) | 1.18 (0.92, 1.5) | 0.92 (0.76, 1.11) | 0.92 (0.75, 1.14) | 1.41 (0.53, 4.19) | 0.93 (0.76, 1.12) | 1.04 (0.82, 1.33) | 1.03 (0.75, 1.44) | 0.89 (0.68, 1.15) | 0.94 (0.78, 1.13) | Saxagliptin | 1.13 (0.86, 1.5) | 0.95 (0.76, 1.19) | 1.11 (0.8, 1.56) | 0.89 (0.71, 1.11) |
| 0.85 (0.66, 1.09) | **0.8 (0.65, 0.98)** | 0.96 (0.85, 1.13) | 0.83 (0.67, 1.04) | 1.52 (0.63, 4.35) | 1.04 (0.8, 1.34) | **0.81 (0.66, 1)** | 0.81 (0.65, 1.02) | 1.25 (0.47, 3.7) | **0.82 (0.66, 1)** | 0.92 (0.72, 1.19) | 0.91 (0.66, 1.28) | 0.78 (0.59, 1.03) | 0.83 (0.67, 1.02) | 0.88 (0.67, 1.17) | Semaglutide | 0.84 (0.67, 1.07) | 0.98 (0.7, 1.4) | **0.79 (0.62, 0.99)** |
| 1.01 (0.83, 1.22) | 0.95 (0.84, 1.06) | 1.14 (0.93, 1.41) | 0.99 (0.86, 1.13) | 1.81 (0.76, 5.12) | 1.23 (1.01, 1.49) | 0.97 (0.86, 1.08) | 0.97 (0.83, 1.12) | 1.48 (0.57, 4.35) | 0.97 (0.86, 1.09) | 1.1 (0.91, 1.32) | 1.08 (0.82, 1.46) | 0.93 (0.74, 1.16) | 0.99 (0.87, 1.1) | 1.05 (0.84, 1.31) | 1.19 (0.94, 1.5) | Sotagliflozin | 1.16 (0.87, 1.59) | 0.93 (0.79, 1.09) |
| 0.87 (0.62, 1.18) | 0.82 (0.61, 1.07) | 0.98 (0.7, 1.36) | 0.85 (0.63, 1.13) | 1.56 (0.63, 4.5) | 1.06 (0.76, 1.45) | 0.83 (0.62, 1.09) | 0.83 (0.61, 1.11) | 1.27 (0.47, 3.83) | 0.84 (0.62, 1.1) | 0.94 (0.68, 1.28) | 0.93 (0.63, 1.37) | 0.8 (0.57, 1.11) | 0.85 (0.63, 1.11) | 0.9 (0.64, 1.25) | 1.02 (0.72, 1.43) | 0.86 (0.63, 1.15) | Trelagliptin | 0.8 (0.59, 1.08) |
| 1.08 (0.89, 1.3) | 1.02 (0.91, 1.14) | 1.22 (1.01, 1.51) | 1.06 (0.93, 1.21) | 1.94 (0.82, 5.47) | 1.32 (1.09, 1.6) | 1.04 (0.93, 1.16) | 1.04 (0.9, 1.2) | 1.59 (0.61, 4.65) | 1.04 (0.93, 1.16) | 1.17 (0.98, 1.41) | 1.15 (0.88, 1.57) | 1 (0.8, 1.24) | 1.05 (0.94, 1.18) | 1.12 (0.9, 1.4) | 1.27 (1.01, 1.61) | 1.07 (0.92, 1.26) | 1.25 (0.93, 1.7) | Vildagliptin |

Note:The bold data represent significant differences.

**Table S7** Comparison of hypoglycemia in patients with type 2 diabetes and chronic kidney disease

| Bexagliflozin | 0.99 (0.58, 1.68) | 1.48 (0.74, 3.12) | 0.95 (0.61, 1.47) | 0.39 (0.01, 4.66) | 1.33 (0.83, 2.11) | 0.99 (0.67, 1.47) | 0.94 (0.55, 1.61) | 0.99 (0.11, 8.77) | 0.99 (0.67, 1.47) | 0.53 (0.09, 3.03) | 1.14 (0.58, 2.27) | 0.99 (0.67, 1.45) | 2.03 (0.6, 8.05) | 0.88 (0.51, 1.5) | 0.96 (0.59, 1.59) | 1.36 (0.76, 2.47) |
| --- | --- | --- | --- | --- | --- | --- | --- | --- | --- | --- | --- | --- | --- | --- | --- | --- |
| 1.01 (0.59, 1.73) | Canagliflozin | 1.5 (0.76, 3.12) | 0.96 (0.63, 1.46) | 0.4 (0.01, 4.74) | 1.35 (0.87, 2.08) | 1 (0.69, 1.45) | 0.95 (0.57, 1.6) | 1.01 (0.11, 8.82) | 1 (0.69, 1.45) | 0.53 (0.09, 3.07) | 1.16 (0.6, 2.27) | 1 (0.69, 1.44) | 2.06 (0.62, 8.07) | 0.89 (0.53, 1.48) | 0.98 (0.61, 1.58) | 1.38 (0.78, 2.46) |
| 0.67 (0.32, 1.36) | 0.67 (0.32, 1.31) | Cotadutide | 0.64 (0.33, 1.18) | 0.26 (0.01, 3.28) | 0.9 (0.45, 1.68) | 0.67 (0.35, 1.2) | 0.63 (0.31, 1.26) | 0.67 (0.07, 6.14) | 0.67 (0.35, 1.19) | 0.35 (0.06, 2.16) | 0.77 (0.33, 1.74) | 0.67 (0.35, 1.18) | 1.37 (0.37, 5.74) | 0.59 (0.31, 1.07) | 0.65 (0.32, 1.26) | 0.92 (0.43, 1.91) |
| 1.06 (0.68, 1.64) | 1.04 (0.69, 1.57) | 1.56 (0.85, 3.05) | Dapagliflozin | 0.41 (0.01, 4.78) | 1.4 (1.08, 1.81) | 1.05 (0.84, 1.3) | 0.99 (0.66, 1.51) | 1.05 (0.12, 8.88) | 1.04 (0.84, 1.29) | 0.55 (0.1, 3.12) | 1.2 (0.67, 2.2) | 1.04 (0.85, 1.27) | 2.15 (0.67, 8.13) | 0.92 (0.61, 1.41) | 1.02 (0.71, 1.48) | 1.44 (0.89, 2.35) |
| 2.56 (0.21, 76.38) | 2.53 (0.21, 75.05) | 3.84 (0.3, 115.81) | 2.41 (0.21, 69.77) | Dapagliflozin_Exenatide | 3.39 (0.29, 99.14) | 2.53 (0.22, 73.53) | 2.4 (0.2, 70.56) | 2.53 (0.22, 75.12) | 2.52 (0.22, 73.37) | 1.39 (0.07, 57.23) | 2.93 (0.24, 88.3) | 2.51 (0.22, 73.03) | 5.38 (0.35, 188.71) | 2.24 (0.19, 65.71) | 2.47 (0.21, 72.04) | 3.48 (0.29, 104.95) |
| 0.75 (0.47, 1.2) | 0.74 (0.48, 1.15) | 1.12 (0.6, 2.2) | **0.71 (0.55, 0.92)** | 0.3 (0.01, 3.46) | Dapagliflozin_Saxagliptin | **0.75 (0.58, 0.97)** | 0.7 (0.46, 1.1) | 0.75 (0.09, 6.44) | **0.74 (0.58, 0.97)** | 0.4 (0.07, 2.23) | 0.86 (0.47, 1.6) | **0.74 (0.58, 0.95)** | 1.53 (0.47, 5.85) | 0.66 (0.42, 1.03) | 0.73 (0.49, 1.09) | 1.02 (0.62, 1.71) |
| 1.01 (0.68, 1.5) | 1 (0.69, 1.44) | 1.49 (0.84, 2.84) | 0.96 (0.77, 1.19) | 0.4 (0.01, 4.6) | 1.34 (1.03, 1.74) | Empagliflozin | 0.94 (0.66, 1.38) | 1 (0.11, 8.57) | 1 (0.9, 1.11) | 0.53 (0.1, 2.95) | 1.15 (0.66, 2.04) | 1 (0.92, 1.07) | 2.05 (0.65, 7.68) | 0.89 (0.61, 1.29) | 0.97 (0.72, 1.35) | 1.37 (0.88, 2.17) |
| 1.07 (0.62, 1.81) | 1.06 (0.63, 1.75) | 1.59 (0.8, 3.28) | 1.01 (0.66, 1.52) | 0.42 (0.01, 4.98) | 1.42 (0.91, 2.18) | 1.06 (0.72, 1.52) | Ertugliflozin | 1.06 (0.12, 9.31) | 1.06 (0.72, 1.51) | 0.56 (0.1, 3.24) | 1.22 (0.63, 2.38) | 1.06 (0.73, 1.5) | 2.17 (0.65, 8.52) | 0.94 (0.55, 1.56) | 1.03 (0.64, 1.66) | 1.45 (0.82, 2.58) |
| 1.01 (0.11, 9.1) | 0.99 (0.11, 8.91) | 1.5 (0.16, 14.2) | 0.95 (0.11, 8.25) | 0.4 (0.01, 4.61) | 1.34 (0.16, 11.7) | 1 (0.12, 8.71) | 0.94 (0.11, 8.47) | Exenatide | 0.99 (0.12, 8.69) | 0.53 (0.03, 8.19) | 1.15 (0.13, 10.73) | 0.99 (0.12, 8.66) | 2.08 (0.18, 25.26) | 0.88 (0.1, 7.94) | 0.97 (0.11, 8.64) | 1.37 (0.15, 12.44) |
| 1.01 (0.68, 1.5) | 1 (0.69, 1.45) | 1.5 (0.84, 2.85) | 0.96 (0.78, 1.19) | 0.4 (0.01, 4.64) | 1.34 (1.04, 1.74) | 1 (0.9, 1.11) | 0.95 (0.66, 1.39) | 1.01 (0.12, 8.55) | Linagliptin | 0.53 (0.1, 2.96) | 1.15 (0.66, 2.05) | 1 (0.93, 1.07) | 2.06 (0.65, 7.72) | 0.89 (0.61, 1.29) | 0.98 (0.72, 1.35) | 1.37 (0.89, 2.17) |
| 1.9 (0.33, 10.99) | 1.88 (0.33, 10.76) | 2.83 (0.46, 17.36) | 1.8 (0.32, 10.09) | 0.72 (0.02, 14.99) | 2.52 (0.45, 14.2) | 1.89 (0.34, 10.41) | 1.78 (0.31, 10.27) | 1.88 (0.12, 29.22) | 1.88 (0.34, 10.39) | Luseogliflozin | 2.17 (0.36, 13.09) | 1.88 (0.34, 10.35) | 3.92 (0.49, 32.88) | 1.67 (0.29, 9.61) | 1.84 (0.32, 10.46) | 2.59 (0.44, 15.11) |
| 0.88 (0.44, 1.73) | 0.87 (0.44, 1.68) | 1.3 (0.58, 3.04) | 0.83 (0.45, 1.5) | 0.34 (0.01, 4.23) | 1.16 (0.62, 2.14) | 0.87 (0.49, 1.52) | 0.82 (0.42, 1.6) | 0.87 (0.09, 7.94) | 0.87 (0.49, 1.52) | 0.46 (0.08, 2.81) | Omarigliptin | 0.86 (0.49, 1.51) | 1.78 (0.49, 7.42) | 0.77 (0.39, 1.5) | 0.85 (0.44, 1.6) | 1.19 (0.58, 2.45) |
| 1.01 (0.69, 1.5) | 1 (0.7, 1.44) | 1.5 (0.85, 2.84) | 0.96 (0.79, 1.18) | 0.4 (0.01, 4.63) | 1.35 (1.05, 1.73) | 1 (0.93, 1.08) | 0.95 (0.67, 1.38) | 1.01 (0.12, 8.58) | 1 (0.93, 1.08) | 0.53 (0.1, 2.96) | 1.16 (0.66, 2.05) | Placebo | 2.06 (0.65, 7.73) | 0.89 (0.61, 1.28) | 0.98 (0.73, 1.34) | 1.38 (0.89, 2.17) |
| 0.49 (0.12, 1.66) | 0.49 (0.12, 1.62) | 0.73 (0.17, 2.72) | 0.47 (0.12, 1.49) | 0.19 (0.01, 2.85) | 0.65 (0.17, 2.11) | 0.49 (0.13, 1.54) | 0.46 (0.12, 1.54) | 0.48 (0.04, 5.42) | 0.49 (0.13, 1.53) | 0.25 (0.03, 2.03) | 0.56 (0.13, 2.02) | 0.49 (0.13, 1.53) | Saxagliptin | 0.43 (0.11, 1.45) | 0.47 (0.12, 1.55) | 0.67 (0.17, 2.3) |
| 1.14 (0.67, 1.96) | 1.13 (0.67, 1.89) | 1.69 (0.93, 3.24) | 1.08 (0.71, 1.64) | 0.45 (0.02, 5.36) | 1.52 (0.97, 2.36) | 1.13 (0.78, 1.65) | 1.07 (0.64, 1.8) | 1.14 (0.13, 9.97) | 1.13 (0.78, 1.64) | 0.6 (0.1, 3.45) | 1.3 (0.67, 2.56) | 1.12 (0.78, 1.63) | 2.32 (0.69, 9.13) | Semaglutide | 1.1 (0.69, 1.79) | 1.55 (0.88, 2.77) |
| 1.04 (0.63, 1.69) | 1.02 (0.63, 1.64) | 1.54 (0.8, 3.11) | 0.98 (0.67, 1.41) | 0.41 (0.01, 4.83) | 1.38 (0.92, 2.03) | 1.03 (0.74, 1.4) | 0.97 (0.6, 1.56) | 1.03 (0.12, 8.99) | 1.03 (0.74, 1.39) | 0.54 (0.1, 3.09) | 1.18 (0.62, 2.25) | 1.02 (0.74, 1.38) | 2.11 (0.64, 8.15) | 0.91 (0.56, 1.46) | Sotagliflozin | 1.41 (0.82, 2.42) |
| 0.74 (0.41, 1.32) | 0.73 (0.41, 1.28) | 1.09 (0.52, 2.35) | 0.7 (0.42, 1.13) | 0.29 (0.01, 3.46) | 0.98 (0.58, 1.61) | 0.73 (0.46, 1.13) | 0.69 (0.39, 1.22) | 0.73 (0.08, 6.49) | 0.73 (0.46, 1.13) | 0.39 (0.07, 2.27) | 0.84 (0.41, 1.72) | 0.73 (0.46, 1.12) | 1.49 (0.43, 6.02) | 0.64 (0.36, 1.14) | 0.71 (0.41, 1.22) | Vildagliptin |

Note:The bold data represent significant differences.

**Table S8** Comparison of cardiovascular death in patients with type 2 diabetes and chronic kidney disease

| Canagliflozin | 1.2 (0.9, 1.62) | 1.25 (0.98, 1.59) | 0.91 (0.65, 1.26) | 1.1 (0.8, 1.52) |
| --- | --- | --- | --- | --- |
| 0.83 (0.62, 1.11) | Linagliptin | 1.04 (0.88, 1.22) | **0.75 (0.57, 1)** | 0.91 (0.7, 1.2) |
| 0.8 (0.63, 1.02) | 0.96 (0.82, 1.14) | Placebo | **0.73 (0.58, 0.91)** | 0.88 (0.71, 1.09) |
| 1.1 (0.79, 1.53) | 1.33 (1, 1.75) | 1.38 (1.1, 1.72) | Semaglutide | 1.21 (0.89, 1.65) |
| 0.91 (0.66, 1.25) | 1.09 (0.84, 1.43) | 1.13 (0.92, 1.41) | 0.83 (0.6, 1.12) | Sotagliflozin |

Note:The bold data represent significant differences.

**Table S9** Comparison of all-cause mortality(≤26weeks) in patients with Type 2 diabetes and chronic kidney disease

|  | **Bexagliflozin** | **Canagliflozin** | **Cotadutide** | **Dapagliflozin** | **Dapagliflozin_Saxagliptin** | **Ertugliflozin** | **Liraglutide** | **Luseogliflozin** | **Omarigliptin** | **Placebo** | **Semaglutide** | **Trelagliptin** | **Vildagliptin** |
| --- | --- | --- | --- | --- | --- | --- | --- | --- | --- | --- | --- | --- | --- |
| Bexagliflozin | Bexagliflozin | 0.52 (0, 78.63) | 1.69 (0.03, 93.44) | 1.66 (0.03, 102.54) | 1.72 (0.02, 125.03) | 2.05 (0.03, 238.65) | 5.59 (0.08, 639.74) | 1.34 (0.02, 169.62) | 1 (0.01, 147.16) | 1 (0.03, 36.72) | 1.72 (0.03, 93.4) | 0.91 (0.01, 131.99) | 0.61 (0.01, 28.28) |
| Canagliflozin | 1.94 (0.01, 284.22) | Canagliflozin | 3.34 (0.07, 169.47) | 3.23 (0.06, 195.31) | 3.33 (0.05, 241.2) | 3.96 (0.06, 458.32) | 10.86 (0.16, 1228.62) | 2.65 (0.03, 314.98) | 1.95 (0.01, 278.4) | 1.93 (0.05, 68.04) | 3.36 (0.06, 177.86) | 1.77 (0.01, 257.07) | 1.18 (0.03, 51.62) |
| Cotadutide | 0.59 (0.01, 29.73) | 0.3 (0.01, 15.34) | Cotadutide | 0.97 (0.07, 13.56) | 1.01 (0.05, 18.01) | 1.15 (0.06, 44.54) | 3.14 (0.19, 117.38) | 0.77 (0.03, 31.42) | 0.59 (0.01, 28.83) | 0.6 (0.1, 2.83) | 1.04 (0.13, 6.08) | 0.54 (0.01, 25.93) | 0.36 (0.04, 2.88) |
| Dapagliflozin | 0.6 (0.01, 34.7) | 0.31 (0.01, 17.57) | 1.03 (0.07, 13.61) | Dapagliflozin | 1.05 (0.11, 8.54) | 1.2 (0.05, 51.74) | 3.24 (0.16, 137.58) | 0.8 (0.03, 36.42) | 0.61 (0.01, 33.84) | 0.61 (0.07, 3.94) | 1.05 (0.07, 14.55) | 0.55 (0.01, 30.47) | 0.37 (0.03, 3.75) |
| Dapagliflozin_Saxagliptin | 0.58 (0.01, 41.14) | 0.3 (0, 20.91) | 0.99 (0.06, 18.4) | 0.95 (0.12, 8.89) | Dapagliflozin_Saxagliptin | 1.16 (0.04, 62.19) | 3.17 (0.12, 163.89) | 0.78 (0.02, 44.46) | 0.58 (0.01, 39.86) | 0.58 (0.05, 5.97) | 1 (0.05, 19.29) | 0.53 (0.01, 35.91) | 0.35 (0.02, 5.24) |
| Ertugliflozin | 0.49 (0, 36.34) | 0.25 (0, 17.86) | 0.87 (0.02, 15.67) | 0.83 (0.02, 18.39) | 0.86 (0.02, 23.82) | Ertugliflozin | 2.75 (0.06, 141.14) | 0.66 (0.01, 37.56) | 0.49 (0, 33.84) | 0.53 (0.02, 4.82) | 0.87 (0.02, 16.75) | 0.45 (0, 30.3) | 0.31 (0.01, 4.41) |
| Liraglutide | 0.18 (0, 12.32) | 0.09 (0, 6.2) | 0.32 (0.01, 5.16) | 0.31 (0.01, 6.12) | 0.32 (0.01, 8.08) | 0.36 (0.01, 17.96) | Liraglutide | 0.24 (0, 12.71) | 0.18 (0, 11.79) | 0.19 (0.01, 1.51) | 0.32 (0.01, 5.41) | 0.16 (0, 10.71) | 0.11 (0, 1.42) |
| Luseogliflozin | 0.74 (0.01, 62.83) | 0.38 (0, 32.05) | 1.3 (0.03, 29.68) | 1.25 (0.03, 34.98) | 1.29 (0.02, 44.84) | 1.52 (0.03, 94.68) | 4.15 (0.08, 249.19) | Luseogliflozin | 0.74 (0.01, 60.24) | 0.78 (0.03, 9.84) | 1.3 (0.03, 31.4) | 0.67 (0.01, 55.48) | 0.46 (0.01, 8.55) |
| Omarigliptin | 1 (0.01, 157.33) | 0.51 (0, 78.86) | 1.7 (0.03, 91.74) | 1.65 (0.03, 103.91) | 1.72 (0.03, 125.88) | 2.03 (0.03, 243.67) | 5.57 (0.08, 647.03) | 1.35 (0.02, 173.53) | Omarigliptin | 0.99 (0.03, 37.07) | 1.72 (0.03, 95.19) | 0.91 (0.01, 140.17) | 0.6 (0.01, 28.11) |
| Placebo | 1 (0.03, 38.53) | 0.52 (0.01, 19.01) | 1.67 (0.35, 10.36) | 1.63 (0.25, 13.91) | 1.72 (0.17, 19.31) | 1.9 (0.21, 54.84) | 5.14 (0.66, 143.55) | 1.28 (0.1, 39.52) | 1.01 (0.03, 36.03) | Placebo | 1.72 (0.29, 11) | 0.93 (0.03, 32.27) | 0.61 (0.15, 2.38) |
| Semaglutide | 0.58 (0.01, 32.5) | 0.3 (0.01, 16.27) | 0.96 (0.16, 7.65) | 0.95 (0.07, 15.28) | 1 (0.05, 19.63) | 1.15 (0.06, 47.93) | 3.13 (0.18, 124.06) | 0.77 (0.03, 33.46) | 0.58 (0.01, 30.81) | 0.58 (0.09, 3.46) | Semaglutide | 0.53 (0.01, 27.87) | 0.35 (0.03, 3.35) |
| Trelagliptin | 1.09 (0.01, 168.18) | 0.57 (0, 85.84) | 1.87 (0.04, 100.01) | 1.8 (0.03, 112.95) | 1.89 (0.03, 136.69) | 2.22 (0.03, 264.38) | 6.12 (0.09, 698.09) | 1.49 (0.02, 183.24) | 1.1 (0.01, 158.35) | 1.08 (0.03, 39.95) | 1.88 (0.04, 105.27) | Trelagliptin | 0.66 (0.01, 30.74) |
| Vildagliptin | 1.65 (0.04, 80.17) | 0.84 (0.02, 39.85) | 2.78 (0.35, 27.01) | 2.7 (0.27, 34.32) | 2.83 (0.19, 46.23) | 3.21 (0.23, 116.59) | 8.75 (0.7, 301.57) | 2.15 (0.12, 82.26) | 1.66 (0.04, 74.51) | 1.63 (0.42, 6.8) | 2.83 (0.3, 28.78) | 1.51 (0.03, 67.08) | Vildagliptin |

**Table S10** Comparison of all-cause mortality(>26weeks) in patients with Type 2 diabetes and chronic kidney disease

|  | **Canagliflozin** | **Dapagliflozin** | **Empagliflozin** | **Ertugliflozin** | **Linagliptin** | **Placebo** | **Saxagliptin** | **Semaglutide** | **Sotagliflozin** |
| --- | --- | --- | --- | --- | --- | --- | --- | --- | --- |
| Canagliflozin | Canagliflozin | 0.59 (0.16, 2.16) | 0.86 (0.64, 1.15) | 1.45 (0.39, 7.09) | 1.15 (0.91, 1.46) | 1.17 (0.97, 1.43) | 0.85 (0.16, 4.03) | 0.95 (0.74, 1.23) | 1.16 (0.89, 1.5) |
| Dapagliflozin | 1.7 (0.46, 6.14) | Dapagliflozin | 1.45 (0.39, 5.29) | 2.49 (0.39, 18.54) | 1.96 (0.54, 7.04) | 2 (0.55, 7.12) | 1.44 (0.18, 10.7) | 1.62 (0.44, 5.84) | 1.97 (0.54, 7.1) |
| Empagliflozin | 1.17 (0.87, 1.57) | 0.69 (0.19, 2.54) | Empagliflozin | 1.69 (0.45, 8.33) | 1.35 (1.04, 1.75) | 1.37 (1.09, 1.72) | 1 (0.19, 4.72) | 1.12 (0.84, 1.48) | 1.36 (1.02, 1.8) |
| Ertugliflozin | 0.69 (0.14, 2.57) | 0.4 (0.05, 2.55) | 0.59 (0.12, 2.21) | Ertugliflozin | 0.8 (0.16, 2.94) | 0.81 (0.17, 2.96) | 0.58 (0.06, 4.46) | 0.66 (0.14, 2.45) | 0.8 (0.16, 2.97) |
| Linagliptin | 0.87 (0.68, 1.1) | 0.51 (0.14, 1.87) | 0.74 (0.57, 0.97) | 1.26 (0.34, 6.13) | Linagliptin | 1.02 (0.89, 1.17) | 0.74 (0.14, 3.47) | 0.83 (0.67, 1.03) | 1 (0.81, 1.25) |
| Placebo | 0.85 (0.7, 1.03) | 0.5 (0.14, 1.82) | 0.73 (0.58, 0.91) | 1.23 (0.34, 5.97) | 0.98 (0.86, 1.12) | Placebo | 0.73 (0.14, 3.38) | 0.81 (0.69, 0.96) | 0.99 (0.83, 1.17) |
| Saxagliptin | 1.17 (0.25, 6.2) | 0.69 (0.09, 5.58) | 1 (0.21, 5.33) | 1.74 (0.22, 16.51) | 1.35 (0.29, 7.11) | 1.38 (0.3, 7.21) | Saxagliptin | 1.12 (0.24, 5.88) | 1.36 (0.29, 7.17) |
| Semaglutide | 1.05 (0.81, 1.35) | 0.62 (0.17, 2.27) | 0.9 (0.68, 1.18) | 1.52 (0.41, 7.4) | 1.21 (0.98, 1.49) | 1.23 (1.05, 1.45) | 0.89 (0.17, 4.19) | Semaglutide | 1.21 (0.96, 1.54) |
| Sotagliflozin | 0.86 (0.67, 1.12) | 0.51 (0.14, 1.86) | 0.74 (0.56, 0.98) | 1.25 (0.34, 6.11) | 1 (0.8, 1.24) | 1.01 (0.85, 1.2) | 0.74 (0.14, 3.47) | 0.82 (0.65, 1.04) | Sotagliflozin |

**Table S11** Comparison of AEs(≤26weeks) in patients with Type 2 diabetes and chronic kidney disease

|  | **Bexagliflozin** | **Canagliflozin** | **Cotadutide** | **Dapagliflozin** | **Dapagliflozin_Exenatide** | **Dapagliflozin_Saxagliptin** | **Ertugliflozin** | **Exenatide** | **Liraglutide** | **Luseogliflozin** | **Omarigliptin** | **Placebo** | **Semaglutide** | **Trelagliptin** | **Vildagliptin** |
| --- | --- | --- | --- | --- | --- | --- | --- | --- | --- | --- | --- | --- | --- | --- | --- |
| Bexagliflozin | Bexagliflozin | 1.01 (0.82, 1.25) | 1.13 (0.91, 1.42) | 0.92 (0.74, 1.14) | 1.67 (0.69, 4.72) | 1.19 (0.95, 1.49) | 0.94 (0.76, 1.15) | 1.37 (0.52, 4) | 1.09 (0.88, 1.34) | 1.07 (0.8, 1.48) | 0.92 (0.72, 1.17) | 0.98 (0.84, 1.14) | 1.18 (0.91, 1.52) | 1.15 (0.85, 1.59) | 0.93 (0.77, 1.12) |
| Canagliflozin | 0.99 (0.8, 1.22) | Canagliflozin | 1.12 (0.9, 1.41) | 0.91 (0.73, 1.13) | 1.66 (0.69, 4.71) | 1.18 (0.94, 1.47) | 0.93 (0.76, 1.13) | 1.35 (0.51, 3.98) | 1.08 (0.87, 1.33) | 1.06 (0.79, 1.46) | 0.91 (0.72, 1.16) | 0.97 (0.83, 1.11) | 1.17 (0.9, 1.5) | 1.14 (0.84, 1.57) | 0.92 (0.76, 1.1) |
| Cotadutide | 0.88 (0.7, 1.1) | 0.89 (0.71, 1.11) | Cotadutide | 0.81 (0.64, 1.01) | 1.47 (0.61, 4.2) | 1.05 (0.82, 1.32) | 0.83 (0.66, 1.02) | 1.2 (0.45, 3.56) | 0.96 (0.76, 1.19) | 0.94 (0.7, 1.31) | 0.81 (0.63, 1.04) | 0.86 (0.72, 1.01) | 1.05 (0.89, 1.18) | 1.02 (0.74, 1.41) | 0.82 (0.67, 0.99) |
| Dapagliflozin | 1.09 (0.87, 1.36) | 1.1 (0.88, 1.37) | 1.23 (0.99, 1.56) | Dapagliflozin | 1.82 (0.78, 5.08) | 1.29 (1.08, 1.54) | 1.02 (0.83, 1.26) | 1.49 (0.57, 4.29) | 1.18 (0.95, 1.47) | 1.16 (0.87, 1.61) | 1 (0.78, 1.28) | 1.06 (0.91, 1.25) | 1.28 (0.99, 1.66) | 1.26 (0.92, 1.74) | 1.01 (0.83, 1.22) |
| Dapagliflozin_Exenatide | 0.6 (0.21, 1.44) | 0.6 (0.21, 1.45) | 0.68 (0.24, 1.64) | 0.55 (0.2, 1.28) | Dapagliflozin_Exenatide | 0.71 (0.25, 1.69) | 0.56 (0.2, 1.34) | 0.82 (0.36, 1.74) | 0.65 (0.23, 1.56) | 0.64 (0.22, 1.59) | 0.55 (0.19, 1.33) | 0.58 (0.21, 1.38) | 0.7 (0.25, 1.72) | 0.69 (0.24, 1.73) | 0.55 (0.2, 1.33) |
| Dapagliflozin_Saxagliptin | 0.84 (0.67, 1.06) | 0.85 (0.68, 1.07) | 0.95 (0.76, 1.22) | 0.77 (0.65, 0.92) | 1.41 (0.59, 3.97) | Dapagliflozin_Saxagliptin | 0.79 (0.64, 0.98) | 1.15 (0.44, 3.36) | 0.92 (0.73, 1.15) | 0.9 (0.67, 1.25) | 0.78 (0.6, 1) | 0.82 (0.69, 0.97) | 0.99 (0.76, 1.29) | 0.97 (0.71, 1.35) | 0.78 (0.64, 0.95) |
| Ertugliflozin | 1.07 (0.87, 1.31) | 1.08 (0.88, 1.32) | 1.21 (0.98, 1.5) | 0.98 (0.8, 1.21) | 1.79 (0.74, 5.05) | 1.27 (1.02, 1.57) | Ertugliflozin | 1.46 (0.55, 4.28) | 1.16 (0.95, 1.42) | 1.14 (0.86, 1.56) | 0.99 (0.78, 1.24) | 1.04 (0.91, 1.19) | 1.26 (0.99, 1.61) | 1.23 (0.91, 1.69) | 0.99 (0.83, 1.18) |
| Exenatide | 0.73 (0.25, 1.94) | 0.74 (0.25, 1.96) | 0.83 (0.28, 2.2) | 0.67 (0.23, 1.74) | 1.22 (0.57, 2.81) | 0.87 (0.3, 2.28) | 0.68 (0.23, 1.81) | Exenatide | 0.79 (0.27, 2.11) | 0.78 (0.26, 2.14) | 0.67 (0.23, 1.8) | 0.71 (0.24, 1.87) | 0.86 (0.29, 2.3) | 0.85 (0.28, 2.31) | 0.68 (0.23, 1.78) |
| Liraglutide | 0.92 (0.74, 1.14) | 0.93 (0.75, 1.15) | 1.04 (0.84, 1.31) | 0.85 (0.68, 1.05) | 1.54 (0.64, 4.37) | 1.09 (0.87, 1.36) | 0.86 (0.71, 1.05) | 1.26 (0.47, 3.7) | Liraglutide | 0.98 (0.74, 1.35) | 0.85 (0.67, 1.07) | 0.9 (0.77, 1.04) | 1.09 (0.84, 1.4) | 1.06 (0.78, 1.46) | 0.85 (0.71, 1.02) |
| Luseogliflozin | 0.94 (0.68, 1.25) | 0.94 (0.69, 1.26) | 1.06 (0.77, 1.43) | 0.86 (0.62, 1.16) | 1.56 (0.63, 4.5) | 1.11 (0.8, 1.5) | 0.88 (0.64, 1.16) | 1.27 (0.47, 3.81) | 1.02 (0.74, 1.36) | Luseogliflozin | 0.86 (0.61, 1.18) | 0.91 (0.69, 1.17) | 1.1 (0.78, 1.52) | 1.08 (0.73, 1.58) | 0.87 (0.64, 1.14) |
| Omarigliptin | 1.08 (0.85, 1.38) | 1.09 (0.86, 1.39) | 1.23 (0.96, 1.59) | 1 (0.78, 1.28) | 1.82 (0.75, 5.17) | 1.29 (1, 1.66) | 1.01 (0.81, 1.28) | 1.48 (0.56, 4.37) | 1.18 (0.93, 1.5) | 1.16 (0.85, 1.63) | Omarigliptin | 1.06 (0.88, 1.28) | 1.28 (0.97, 1.69) | 1.25 (0.9, 1.76) | 1 (0.81, 1.25) |
| Placebo | 1.03 (0.88, 1.2) | 1.03 (0.9, 1.21) | 1.16 (0.99, 1.39) | 0.94 (0.8, 1.1) | 1.72 (0.72, 4.83) | 1.22 (1.03, 1.44) | 0.96 (0.84, 1.1) | 1.4 (0.54, 4.09) | 1.11 (0.96, 1.29) | 1.1 (0.86, 1.46) | 0.95 (0.78, 1.14) | Placebo | 1.21 (0.98, 1.48) | 1.18 (0.9, 1.57) | 0.95 (0.85, 1.06) |
| Semaglutide | 0.85 (0.66, 1.1) | 0.85 (0.67, 1.11) | 0.96 (0.84, 1.13) | 0.78 (0.6, 1.01) | 1.42 (0.58, 4.06) | 1.01 (0.77, 1.31) | 0.79 (0.62, 1.02) | 1.16 (0.43, 3.44) | 0.92 (0.72, 1.19) | 0.91 (0.66, 1.29) | 0.78 (0.59, 1.03) | 0.83 (0.67, 1.02) | Semaglutide | 0.98 (0.7, 1.39) | 0.78 (0.62, 0.99) |
| Trelagliptin | 0.87 (0.63, 1.18) | 0.87 (0.64, 1.19) | 0.98 (0.71, 1.35) | 0.8 (0.57, 1.09) | 1.45 (0.58, 4.2) | 1.03 (0.74, 1.41) | 0.81 (0.59, 1.1) | 1.18 (0.43, 3.56) | 0.94 (0.68, 1.28) | 0.93 (0.63, 1.37) | 0.8 (0.57, 1.11) | 0.85 (0.64, 1.11) | 1.02 (0.72, 1.43) | Trelagliptin | 0.8 (0.59, 1.07) |
| Vildagliptin | 1.08 (0.89, 1.3) | 1.09 (0.91, 1.32) | 1.22 (1.01, 1.5) | 0.99 (0.82, 1.2) | 1.81 (0.75, 5.09) | 1.28 (1.05, 1.57) | 1.01 (0.85, 1.21) | 1.48 (0.56, 4.31) | 1.17 (0.98, 1.41) | 1.15 (0.88, 1.57) | 1 (0.8, 1.24) | 1.05 (0.94, 1.18) | 1.27 (1.01, 1.6) | 1.25 (0.93, 1.69) | Vildagliptin |

**Table S12** Comparison of AEs (>26weeks) in patients with Type 2 diabetes and chronic kidney disease

|  | **Canagliflozin** | **Dapagliflozin** | **Empagliflozin** | **Ertugliflozin** | **Linagliptin** | **Placebo** | **Saxagliptin** | **Sotagliflozin** |
| --- | --- | --- | --- | --- | --- | --- | --- | --- |
| Canagliflozin | Canagliflozin | 1.06 (0.98, 1.16) | 1.02 (0.99, 1.05) | 1.02 (0.93, 1.13) | 1.02 (0.99, 1.06) | 1.04 (1.01, 1.06) | 1.11 (0.92, 1.34) | 1.05 (0.94, 1.19) |
| Dapagliflozin | 0.94 (0.86, 1.02) | Dapagliflozin | 0.96 (0.88, 1.04) | 0.96 (0.84, 1.09) | 0.97 (0.88, 1.04) | 0.98 (0.89, 1.05) | 1.04 (0.85, 1.28) | 0.99 (0.86, 1.14) |
| Empagliflozin | 0.98 (0.95, 1.01) | 1.04 (0.96, 1.14) | Empagliflozin | 1 (0.91, 1.1) | 1 (0.97, 1.03) | 1.02 (1, 1.03) | 1.08 (0.9, 1.31) | 1.03 (0.93, 1.17) |
| Ertugliflozin | 0.98 (0.89, 1.08) | 1.04 (0.92, 1.18) | 1 (0.91, 1.1) | Ertugliflozin | 1.01 (0.91, 1.11) | 1.02 (0.92, 1.12) | 1.09 (0.88, 1.34) | 1.03 (0.89, 1.2) |
| Linagliptin | 0.98 (0.94, 1.01) | 1.04 (0.96, 1.14) | 1 (0.97, 1.03) | 0.99 (0.9, 1.1) | Linagliptin | 1.01 (0.99, 1.04) | 1.08 (0.9, 1.31) | 1.03 (0.92, 1.17) |
| Placebo | 0.97 (0.94, 0.99) | 1.02 (0.95, 1.12) | 0.99 (0.97, 1) | 0.98 (0.9, 1.09) | 0.99 (0.96, 1.01) | Placebo | 1.07 (0.89, 1.29) | 1.02 (0.91, 1.15) |
| Saxagliptin | 0.9 (0.75, 1.09) | 0.96 (0.78, 1.18) | 0.92 (0.76, 1.11) | 0.92 (0.74, 1.14) | 0.92 (0.76, 1.12) | 0.94 (0.77, 1.13) | Saxagliptin | 0.95 (0.76, 1.19) |
| Sotagliflozin | 0.95 (0.84, 1.06) | 1.01 (0.87, 1.16) | 0.97 (0.86, 1.08) | 0.97 (0.83, 1.12) | 0.97 (0.86, 1.09) | 0.98 (0.87, 1.1) | 1.05 (0.84, 1.31) | Sotagliflozin |

**Table S13** Comparison of hypoglycemia (≤26weeks) in patients with Type 2 diabetes and chronic kidney disease

|  | **Bexagliflozin** | **Cotadutide** | **Dapagliflozin** | **Dapagliflozin_Exenatide** | **Dapagliflozin_Saxagliptin** | **Ertugliflozin** | **Exenatide** | **Luseogliflozin** | **Omarigliptin** | **Placebo** | **Semaglutide** | **Vildagliptin** |
| --- | --- | --- | --- | --- | --- | --- | --- | --- | --- | --- | --- | --- |
| Bexagliflozin | Bexagliflozin | 1.15 (0.57, 2.51) | 1.09 (0.67, 1.76) | 0.46 (0.02, 5.64) | 1.43 (0.88, 2.3) | 1.07 (0.61, 1.91) | 1.16 (0.13, 10.49) | 0.53 (0.09, 3.07) | 1.14 (0.58, 2.28) | 0.99 (0.67, 1.46) | 0.36 (0.1, 1.09) | 1.36 (0.76, 2.48) |
| Cotadutide | 0.87 (0.4, 1.77) | Cotadutide | 0.94 (0.46, 1.82) | 0.39 (0.01, 5.02) | 1.24 (0.6, 2.38) | 0.93 (0.42, 1.93) | 1 (0.1, 9.56) | 0.45 (0.07, 2.79) | 0.99 (0.41, 2.26) | 0.86 (0.43, 1.54) | 0.32 (0.09, 0.8) | 1.18 (0.53, 2.5) |
| Dapagliflozin | 0.92 (0.57, 1.49) | 1.06 (0.55, 2.19) | Dapagliflozin | 0.42 (0.01, 4.9) | 1.31 (1.01, 1.71) | 0.98 (0.6, 1.64) | 1.06 (0.12, 9.16) | 0.48 (0.08, 2.75) | 1.05 (0.56, 1.98) | 0.91 (0.68, 1.19) | 0.33 (0.09, 0.97) | 1.25 (0.74, 2.13) |
| Dapagliflozin_Exenatide | 2.18 (0.18, 65.71) | 2.55 (0.2, 78.25) | 2.37 (0.2, 69.22) | Dapagliflozin_Exenatide | 3.11 (0.26, 91.37) | 2.35 (0.19, 70.11) | 2.51 (0.21, 75.48) | 1.17 (0.06, 49.64) | 2.51 (0.2, 77.02) | 2.15 (0.18, 63.17) | 0.79 (0.05, 26.9) | 2.98 (0.24, 90.09) |
| Dapagliflozin_Saxagliptin | 0.7 (0.43, 1.13) | 0.81 (0.42, 1.68) | 0.76 (0.58, 0.99) | 0.32 (0.01, 3.79) | Dapagliflozin_Saxagliptin | 0.75 (0.46, 1.25) | 0.81 (0.09, 7.12) | 0.37 (0.06, 2.1) | 0.8 (0.43, 1.5) | 0.69 (0.52, 0.91) | 0.26 (0.07, 0.74) | 0.95 (0.57, 1.62) |
| Ertugliflozin | 0.93 (0.52, 1.64) | 1.08 (0.52, 2.36) | 1.02 (0.61, 1.67) | 0.43 (0.01, 5.25) | 1.33 (0.8, 2.17) | Ertugliflozin | 1.08 (0.12, 9.9) | 0.49 (0.08, 2.88) | 1.07 (0.53, 2.15) | 0.92 (0.6, 1.38) | 0.34 (0.09, 1.02) | 1.27 (0.69, 2.34) |
| Exenatide | 0.86 (0.1, 7.95) | 1 (0.1, 9.81) | 0.94 (0.11, 8.33) | 0.4 (0.01, 4.68) | 1.23 (0.14, 11.05) | 0.92 (0.1, 8.64) | Exenatide | 0.45 (0.03, 7.08) | 0.99 (0.11, 9.48) | 0.85 (0.1, 7.66) | 0.31 (0.03, 3.51) | 1.18 (0.13, 11.08) |
| Luseogliflozin | 1.9 (0.33, 11.11) | 2.21 (0.36, 13.98) | 2.08 (0.36, 11.83) | 0.85 (0.02, 17.46) | 2.72 (0.48, 15.43) | 2.04 (0.35, 12.02) | 2.22 (0.14, 34.13) | Luseogliflozin | 2.18 (0.36, 13.39) | 1.88 (0.34, 10.49) | 0.68 (0.08, 5.11) | 2.6 (0.44, 15.45) |
| Omarigliptin | 0.87 (0.44, 1.72) | 1.01 (0.44, 2.41) | 0.95 (0.51, 1.77) | 0.4 (0.01, 4.99) | 1.25 (0.66, 2.32) | 0.94 (0.46, 1.89) | 1.01 (0.11, 9.43) | 0.46 (0.07, 2.78) | Omarigliptin | 0.86 (0.49, 1.5) | 0.32 (0.08, 1.02) | 1.19 (0.58, 2.44) |
| Placebo | 1.01 (0.69, 1.5) | 1.17 (0.65, 2.3) | 1.1 (0.84, 1.46) | 0.47 (0.02, 5.51) | 1.45 (1.1, 1.91) | 1.08 (0.72, 1.67) | 1.18 (0.13, 10.29) | 0.53 (0.1, 2.98) | 1.16 (0.67, 2.05) | Placebo | 0.37 (0.1, 1.03) | 1.38 (0.89, 2.18) |
| Semaglutide | 2.75 (0.92, 10.27) | 3.16 (1.25, 10.91) | 2.99 (1.03, 10.9) | 1.27 (0.04, 20.13) | 3.91 (1.36, 14.24) | 2.95 (0.98, 11.16) | 3.25 (0.28, 38.53) | 1.46 (0.2, 12.11) | 3.15 (0.98, 12.48) | 2.7 (0.97, 9.61) | Semaglutide | 3.75 (1.23, 14.31) |
| Vildagliptin | 0.73 (0.4, 1.32) | 0.85 (0.4, 1.88) | 0.8 (0.47, 1.34) | 0.34 (0.01, 4.18) | 1.05 (0.62, 1.75) | 0.79 (0.43, 1.46) | 0.85 (0.09, 7.87) | 0.39 (0.06, 2.28) | 0.84 (0.41, 1.72) | 0.72 (0.46, 1.12) | 0.27 (0.07, 0.81) | Vildagliptin |

**Table S14** Comparison of hypoglycemia (>26weeks) in patients with Type 2 diabetes and chronic kidney disease

|  | **Canagliflozin** | **Dapagliflozin** | **Empagliflozin** | **Ertugliflozin** | **Linagliptin** | **Placebo** | **Saxagliptin** | **Semaglutide** | **Sotagliflozin** |
| --- | --- | --- | --- | --- | --- | --- | --- | --- | --- |
| Canagliflozin | Canagliflozin | 0.83 (0.53, 1.31) | 1.01 (0.69, 1.45) | 0.95 (0.57, 1.59) | 1 (0.69, 1.45) | 1 (0.7, 1.44) | 2.08 (0.63, 8.26) | 1.02 (0.59, 1.76) | 0.98 (0.61, 1.58) |
| Dapagliflozin | 1.2 (0.76, 1.9) | Dapagliflozin | 1.21 (0.9, 1.6) | 1.14 (0.72, 1.81) | 1.21 (0.9, 1.59) | 1.2 (0.91, 1.58) | 2.5 (0.77, 9.69) | 1.23 (0.75, 2) | 1.18 (0.78, 1.78) |
| Empagliflozin | 1 (0.69, 1.44) | 0.83 (0.62, 1.11) | Empagliflozin | 0.94 (0.66, 1.38) | 1 (0.9, 1.11) | 1 (0.92, 1.07) | 2.07 (0.66, 7.86) | 1.01 (0.67, 1.54) | 0.97 (0.72, 1.34) |
| Ertugliflozin | 1.05 (0.63, 1.75) | 0.88 (0.55, 1.38) | 1.06 (0.72, 1.52) | Ertugliflozin | 1.06 (0.72, 1.52) | 1.05 (0.72, 1.51) | 2.19 (0.66, 8.65) | 1.07 (0.62, 1.85) | 1.03 (0.64, 1.66) |
| Linagliptin | 1 (0.69, 1.44) | 0.83 (0.63, 1.11) | 1 (0.9, 1.11) | 0.95 (0.66, 1.38) | Linagliptin | 1 (0.93, 1.07) | 2.07 (0.66, 7.86) | 1.02 (0.67, 1.54) | 0.98 (0.72, 1.35) |
| Placebo | 1 (0.7, 1.44) | 0.83 (0.63, 1.1) | 1 (0.93, 1.08) | 0.95 (0.66, 1.38) | 1 (0.93, 1.08) | Placebo | 2.08 (0.66, 7.87) | 1.02 (0.68, 1.53) | 0.98 (0.73, 1.34) |
| Saxagliptin | 0.48 (0.12, 1.6) | 0.4 (0.1, 1.29) | 0.48 (0.13, 1.52) | 0.46 (0.12, 1.51) | 0.48 (0.13, 1.51) | 0.48 (0.13, 1.51) | Saxagliptin | 0.49 (0.12, 1.65) | 0.47 (0.12, 1.54) |
| Semaglutide | 0.98 (0.57, 1.69) | 0.81 (0.5, 1.33) | 0.99 (0.65, 1.49) | 0.93 (0.54, 1.61) | 0.98 (0.65, 1.48) | 0.98 (0.65, 1.47) | 2.04 (0.61, 8.14) | Semaglutide | 0.96 (0.58, 1.59) |
| Sotagliflozin | 1.02 (0.63, 1.64) | 0.85 (0.56, 1.28) | 1.03 (0.74, 1.4) | 0.97 (0.6, 1.56) | 1.03 (0.74, 1.39) | 1.02 (0.75, 1.38) | 2.12 (0.65, 8.33) | 1.04 (0.63, 1.72) | Sotagliflozin |
