# Supplementary material for: Efficacy and safety of novel antidiabetic drugs in patients with type 2 diabetes and chronic kidney disease: a network meta-analysis
Source: Front Endocrinol (Lausanne). 2026 Mar 31;17:1750615. doi: 10.3389/fendo.2026.1750615 (PMC13076170; doi:10.3389/fendo.2026.1750615)
Supplement: Supplementary Table 1 — Search strategy. [file DataSheet1.docx]

**(a)**

**
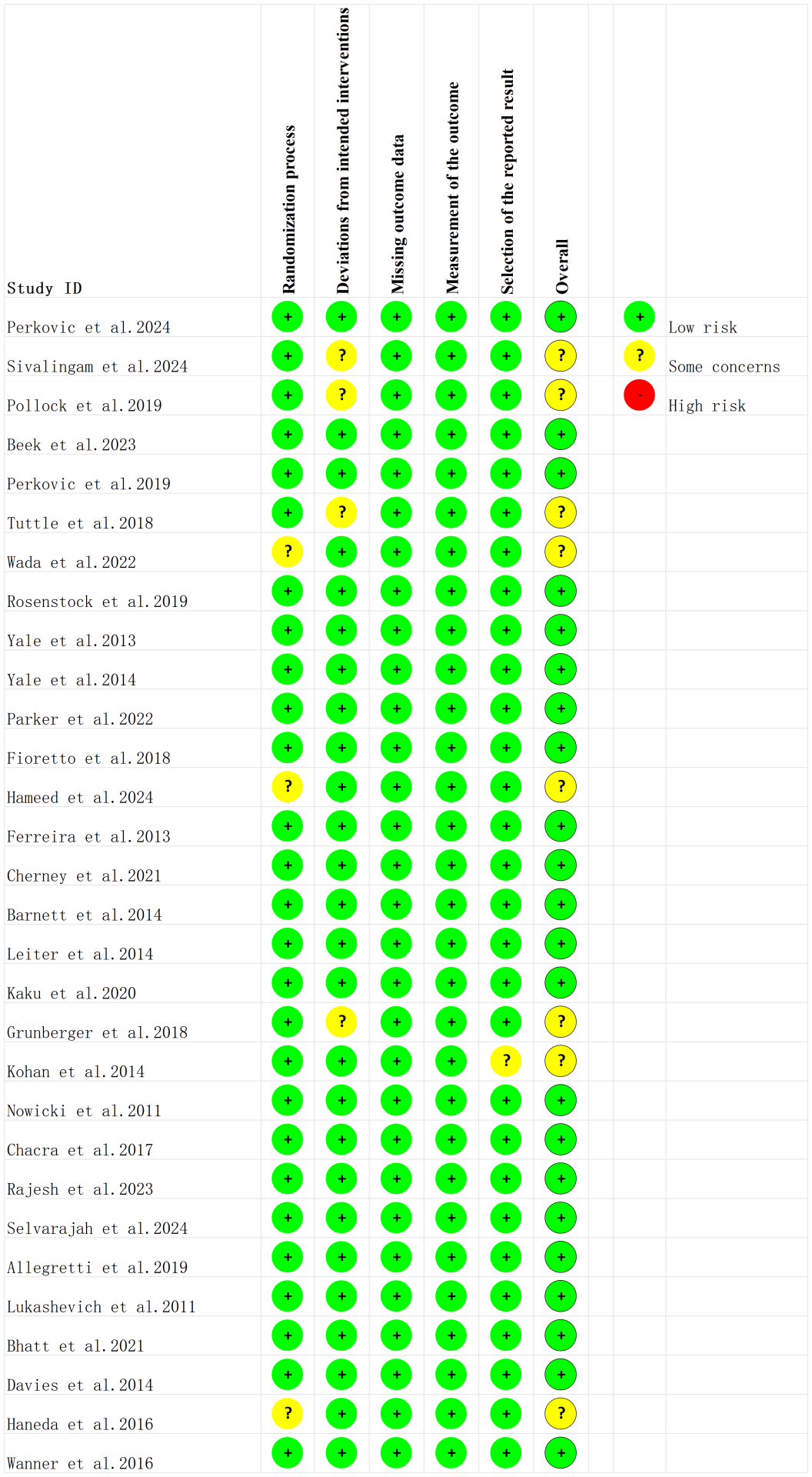
**

**(b)**

**
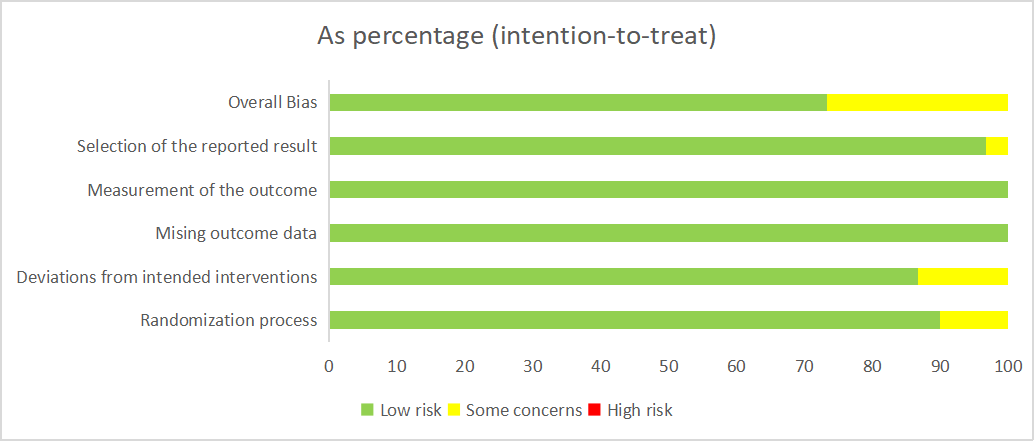
**

**Figure S1** Risk of bias assessment graph

**
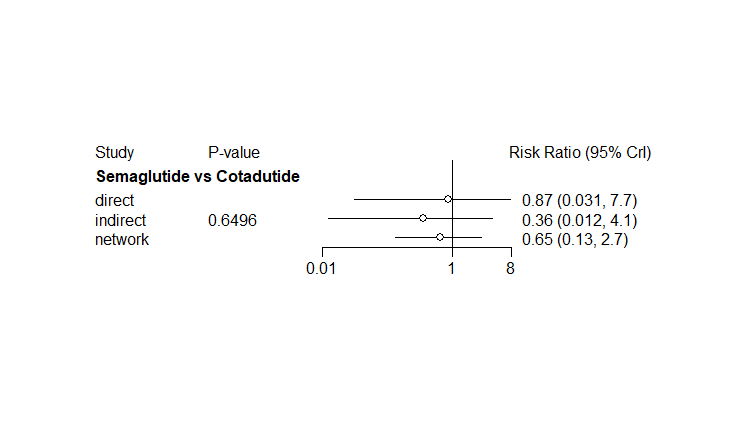
**

**Figure S2** Node-splitting analysis for all-cause mortality

**
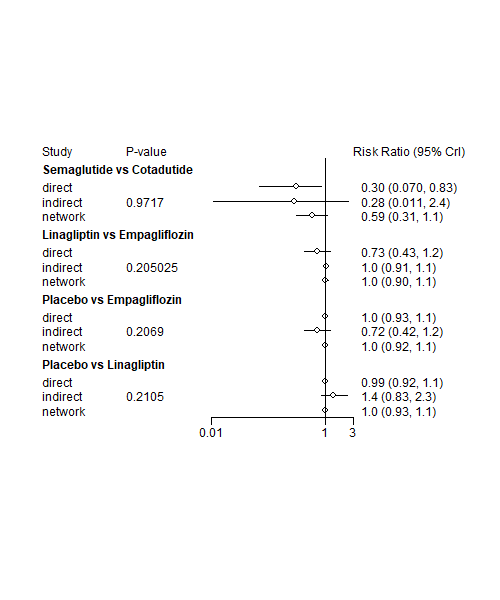
**

**Figure S3** Node-splitting analysis for hypoglycemia

**
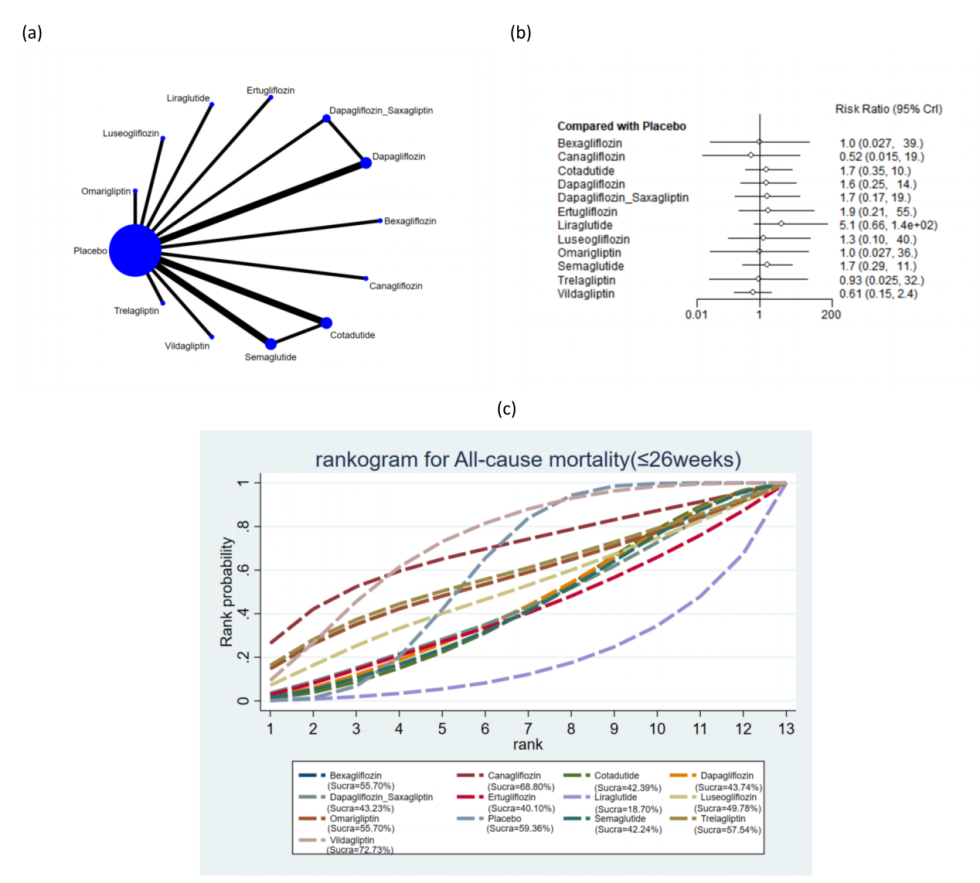
**

**Figure S4** All-cause mortality(≤26weeks)

**
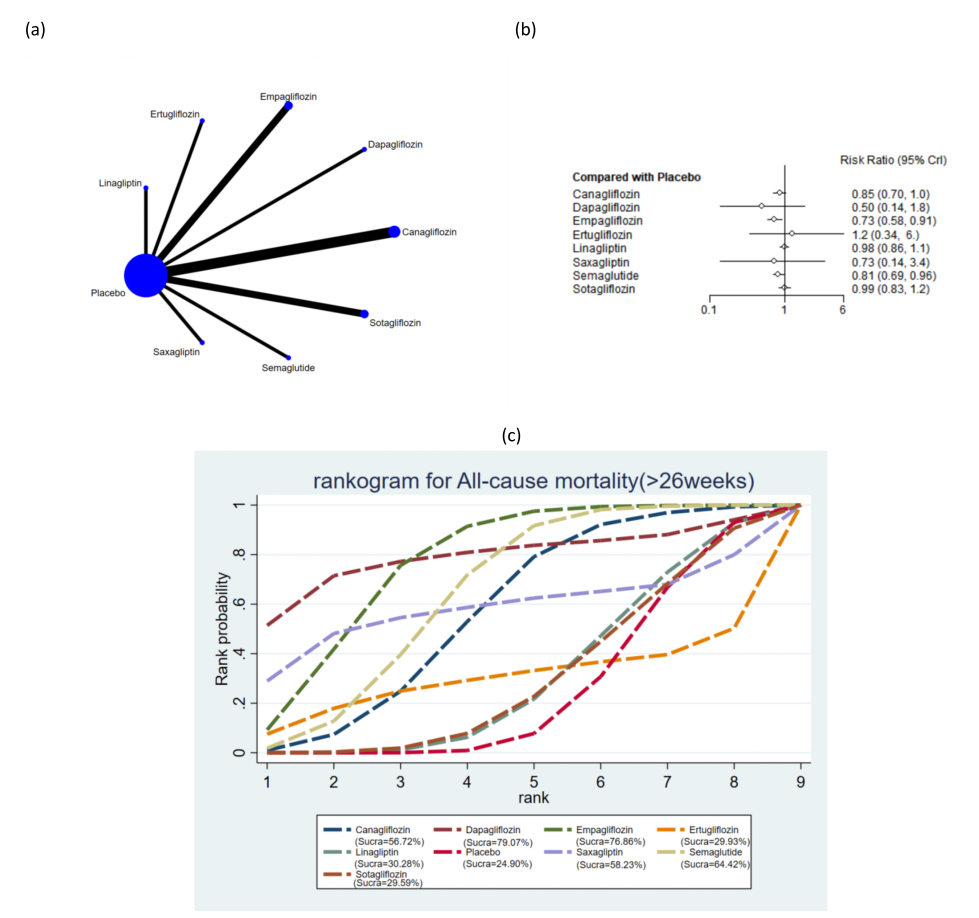
**

**Figure S5** All-cause mortality(>26weeks)

**
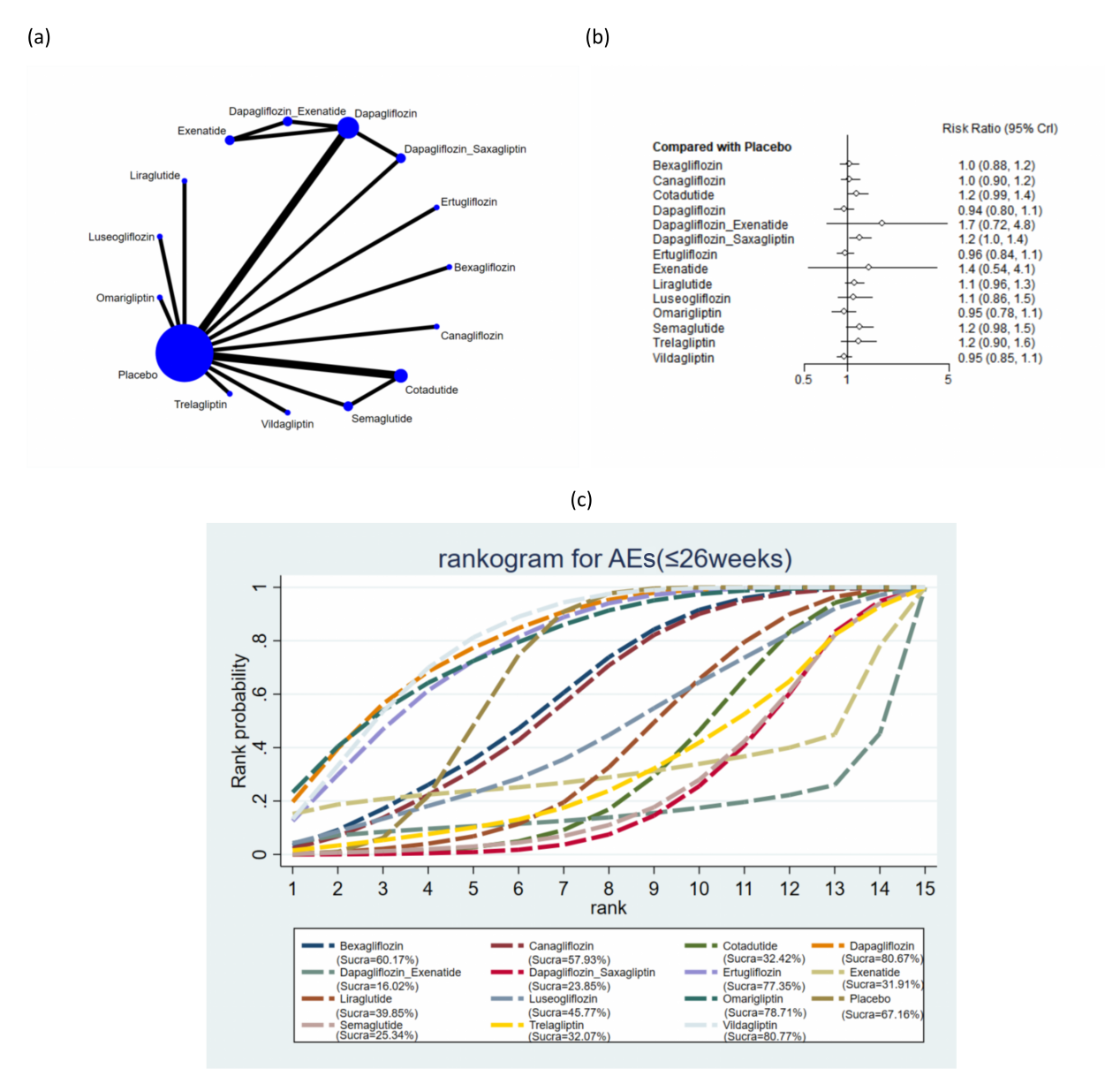
**

**Figure S6** AEs(≤26weeks)

**
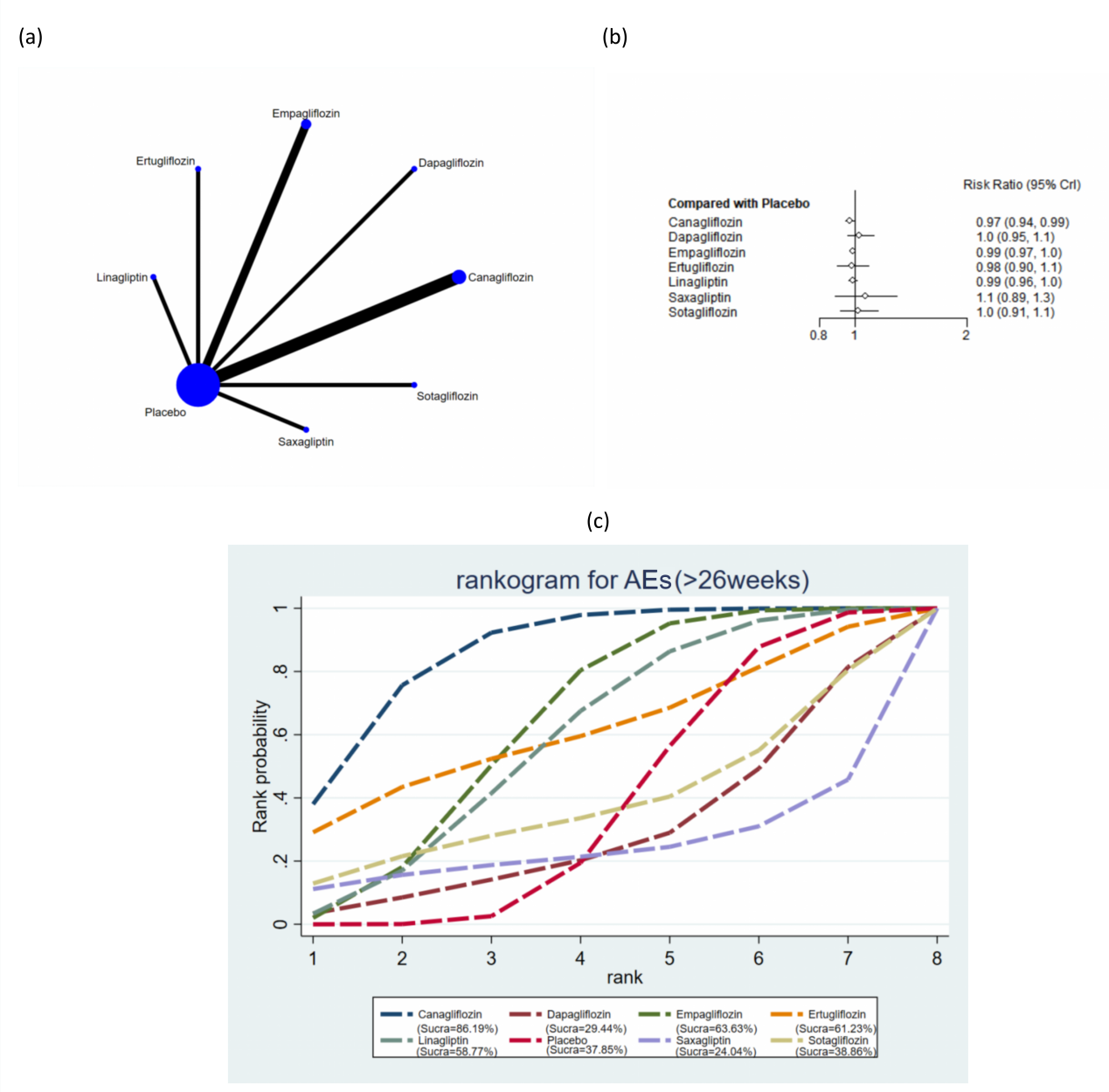
**

**Figure S7** AEs (>26weeks)

**
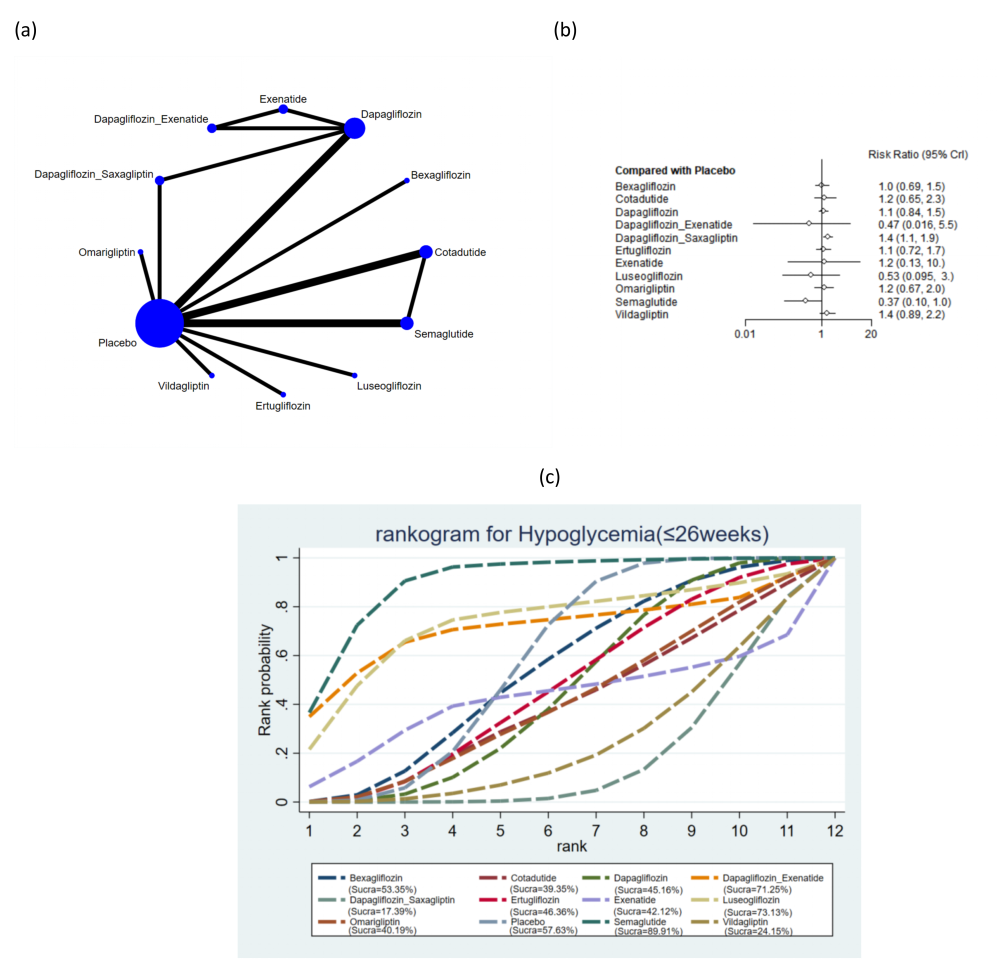
**

**Figure S8** Hypoglycemia(≤26weeks)

**
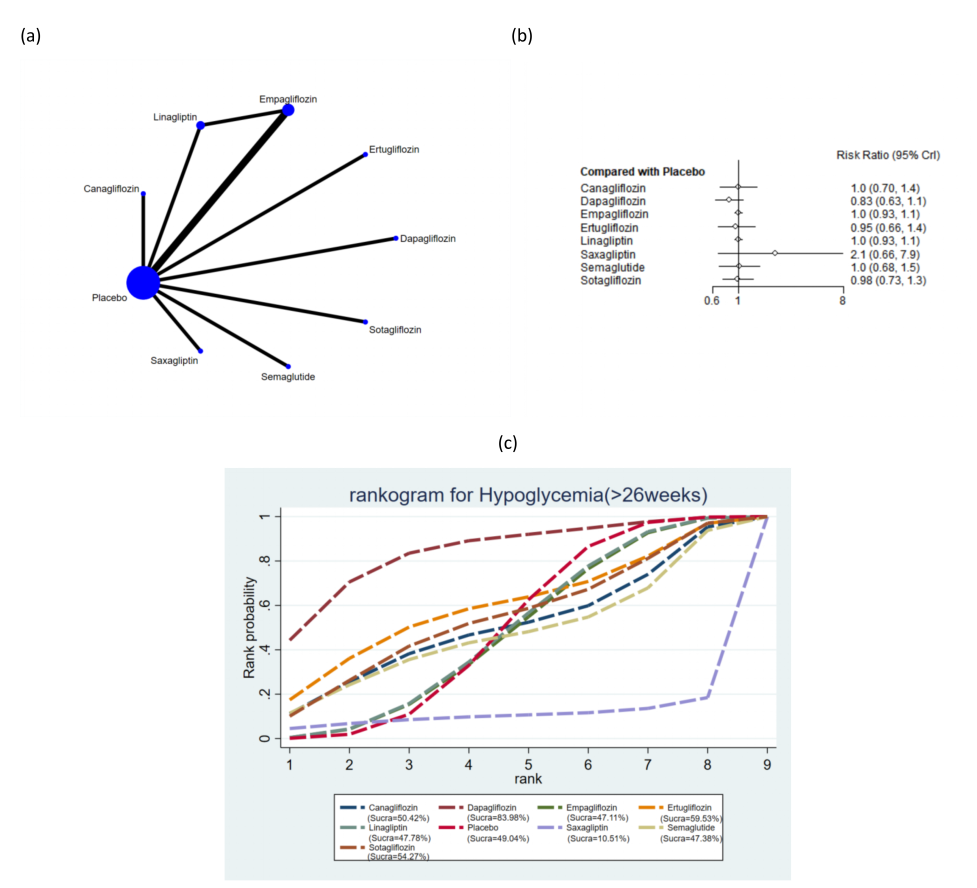
**

**Figure S9** Hypoglycemia(>26weeks)
